# Fermented Soy-Derived Bioactive Peptides Selected by a Molecular Docking Approach Show Antioxidant Properties Involving the Keap1/Nrf2 Pathway

**DOI:** 10.3390/antiox9121306

**Published:** 2020-12-19

**Authors:** Federica Tonolo, Laura Moretto, Alessandro Grinzato, Federico Fiorese, Alessandra Folda, Valeria Scalcon, Stefania Ferro, Giorgio Arrigoni, Marco Bellamio, Emiliano Feller, Alberto Bindoli, Oriano Marin, Maria Pia Rigobello

**Affiliations:** 1 Department of Biomedical Sciences, University of Padova, via Ugo Bassi 58/b, 35131 Padova, Italy; federica.tonolo@phd.unipd.it (F.T.); laura.moretto@unipd.it (L.M.); alessandro.grinzato@phd.unipd.it (A.G.); federico.fiorese.1@gmail.com (F.F.); alessandra.folda.1@unipd.it (A.F.); valeria.scalcon@unipd.it (V.S.); stefania.ferro.1@unipd.it (S.F.); giorgio.arrigoni@unipd.it (G.A.); 2 CRIBI, Biotechnology Center, University of Padova, 35121 Padova, Italy; 3 Proteomics Center, University of Padova and Azienda Ospedaliera di Padova, 35129 Padova, Italy; 4 Centrale del Latte di Vicenza, 36100 Vicenza, Italy; bellamio@centralelatte.vicenza.it (M.B.); feller@centralelatte.vicenza.it (E.F.); 5 Institute of Neuroscience (CNR), viale G. Colombo 3, 35131 Padova, Italy; alberto.bindoli@bio.unipd.it

**Keywords:** antioxidants, Keap1/Nrf2, bioactive peptides, fermented soy, molecular mechanism

## Abstract

Bioactive peptides are a group of molecules with health beneficial properties, deriving from food matrices. They are protein fragments consisting of 2–20 amino acids that can be released by microbial fermentation, food processing and gastrointestinal digestion. Once hydrolyzed from their native proteins, they can have different functions including antioxidant activity, which is important for cell protection by oxidant agents. In this work, fermented soy products were digested in vitro in order to improve the release of bioactive peptides. These were extracted, purified and analyzed in vitro and in a cellular model to assess their antioxidant activity. Peptide sequences were identified by LC-MS/MS analysis and a molecular docking approach was used to predict their ability to interact with Keap1, one of the key proteins of the Keap1/Nrf2 pathway, the major system involved in redox regulation. Peptides showing a high score of interaction were selected and tested for their antioxidant properties in a cellular environment using the Caco-2 cell line and examined for their capability to defend cells against oxidative stress. Our results indicate that several of the selected peptides were indeed able to activate the Keap1/Nrf2 pathway with the consequent overexpression of antioxidant and phase II enzymes.

## 1. Introduction

Soybean (*Glycine Max*) has been cultivated for at least 5000 years. In Asian countries, many fermented soy products are largely consumed, while in Western countries, soybean has received increased interest over the years. Soy-based products are valid alternatives for people with lactose intolerance or a milk allergy and are important in vegetarian and vegan diets, although this food can cause allergic reactions in some subjects [1,2]. The principal components of soybean are proteins (40%), with two major fractions represented by β-conglycinin (7S) and glycinin (11S). Other constituents are lipids, including polyunsaturated fatty acids, carbohydrates, vitamins and minerals. Interestingly, isoflavones (daidzein, glycitein and genistein), phytic acid, phytoalexins, saponins and lectins are an example of soybean molecules endowed with biological activity. Some of these molecules, although present in low quantities, can lead to health benefits for humans, such as in chronic diseases [3,4]. 

Bioactive peptides are an important group of molecules acting in human metabolism. In general, bioactive peptides are formed by 2 to 20 amino acidic residues, originating from animal (milk, eggs and meat) or plant sources, such as soybean or cereals. Enzymatic hydrolysis, fermentation, food processing and gastrointestinal digestion are the main mechanisms by which bioactive peptides can be generated [5]. Once they are released from the native protein, bioactive peptides display antihypertensive, immunomodulatory, anti-cancer, anti-diabetic and antioxidant properties. The latter is important for protection of the body against free radical attack [6]. 

Fermentation is a well-studied process involved in the beneficial effects of some foods. Microorganisms used in food technology are generally lactic acid bacteria, such as the genera *Leuconostoc*, *Streptococcus* and *Lactobacillus* [7]. These bacteria have been selected for soy fermentation because they are able to digest raffinose and stachyose, oligosaccharides that are not hydrolyzed by human enzymes [8]. The use of different fermenting microorganisms can have an important impact on the properties of the final product, such as the generation of bioactive compounds [9]. Besides being generated directly by the microorganisms used for fermentation, bioactive peptides can also be released during the gastrointestinal digestion process. For this reason, we exploited an in vitro gastrointestinal digestion protocol, a technique used also for other purposes, for example, to determine the bioavailability and absorption of different compounds [6,10,11]. Gastrointestinal digestion and food processing factors such as high temperature can influence the structure of peptides; however, Singh B.P. and Vij S. (2018) demonstrated that peptides derived from a fermented soy beverage were stable in vitro under these conditions [12].

In our previous work, fermented soy products were studied and we attributed their increased antioxidant activity at the end of shelf life to the generation of bioactive peptides [13]. The objective of this paper was to extract and identify peptides with potential antioxidant activities released from the digestion of a fermented soybean product. An in vitro method with optimized INFOGEST protocol was used in order to mimic food digestion in the three sequential phases: oral, gastric and intestinal. This technique takes into account many factors such as type of enzymes and related optimal conditions, pH of each phase, salt concentrations and digestion times. Different combinations of enzymes can generate bioactive peptides, with pepsin, trypsin, chymotrypsin and papain being the enzymes mostly used for this purpose [6,10,11]. In this work, fermented soy products were digested with sequential application of pepsin and pancreatin. Peptides generated by this process were purified by reversed-phase high-performance liquid chromatography (RP-HPLC) and identified by LC-MS/MS analysis and database search. Screening for antioxidant activity was carried out in silico using an innovative approach: molecular docking was used to predict the interaction of the peptides with Keap1 protein. The latter is a key factor involved in the regulation of the Keap1/Nrf2 pathway, the major system that modulates the cellular antioxidant response. Under normal conditions, nuclear factor erythroid 2-related factor 2 (Nrf2) is usually sequestered by Keap1 in the cytosol and is sent, after ubiquitination, to the proteasome. Once Keap1 is modified by electrophiles or oxidants, Nrf2 is able to migrate to the nucleus, activating transcription of antioxidant genes. In addition, other molecules such as antioxidant bioactive peptides can induce this process. The Nrf2–antioxidant response element (ARE) complex is involved in the activation of the expression of genes encoding for enzymes involved in the protection of cells against oxidants, such as glutathione peroxidase (Gpx), thioredoxin reductase 1 (TrxR1) or NADPH: quinone oxidoreductase 1 (NQO1) [14,15,16]. The peptides selected by the molecular docking approach were subsequently synthesized and tested for their antioxidant properties in vitro and in a cellular model based on the Caco-2 cell line. Finally, biochemical assays were performed to further investigate their molecular mechanisms of action.

## 2. Materials and Methods

### 2.1. Chemicals and Consumables

(ABTS) 2,2′-azinobis(3-ethylbenzothiazoline 6-sulfonate), 1,1-diphenyl-2-picrylhydrazyl (DPPH), acetonitrile, ammonium carbonate ((NH_4_)_2_CO_3_), butylated hydroxytoluene (BHT), calcium chloride dihydrate (CaCl_2_(H_2_O)_2_), Coomassie Brilliant Blue G-250, cumene hydroperoxide (CHP), dimethyl sulfoxide (DMSO), Folin-Ciocalteau reagent, hydrogen chloride (HCl), isopropanol, IGEPAL CA-630, hemin, 3-(4,5-dimethylthiazolyl-2)-2,5-diphenyl tetrazolium bromide (MTT), magnesium chloride hexahydrate (MgCl_2_ (H_2_O)_6_), n-butanol, pepsin (P6887), pancreatin (P3292), phosphotungstic acid (PTA), potassium chloride (KCl), potassium dihydrogen phosphate (KH_2_PO_4_), potassium persulfate, sodium bicarbonate (NaHCO_3_), sodium carbonate (Na_2_CO_3_), sodium chloride (NaCl), sodium hydroxide (NaOH), sulphuric acid (H_2_SO_4_), *tert*-butyl hydroperoxide (TbOOH), thiobarbituric acid (TBA), trifluoroacetic acid (TFA), Trolox C and Corning Incorporated Transwell^®^ 12-well plates were purchased from Merck (Darmstadt, Germany). Ethanol was purchased from Carlo Erba Reagents S.r.l. (Cornaredo, MI, Italy). CM-H_2_DCFDA was purchased from Thermo Fisher Scientific (Waltham, MA, USA). Dulbecco’s Modified Eagle’s Medium (DMEM) + Glutamax, trypsin + Ethylenediaminetetraacetic acid (EDTA) (0.25%) and fetal bovine serum (FBS) were purchased from Gibco (Thermo Fisher Scientific, Waltham, MA, USA). Primary mouse monoclonal antibodies for Nrf2, TrxR1, superoxide dismutase 1 (SOD1), glutathione reductase (GR), NQO1, GAPDH and Proliferating Cell Nuclear Antigen (PCNA) and goat anti-mouse secondary antibody were purchased from Santa Cruz Biotechnology Inc. (California, USA). For peptide synthesis, the N-α- fluorenylmethyloxycarbonyl (Fmoc) L-amino acids, p-benzyloxybenzyl alcohol resins (Wang resins) and formic acid were all obtained from Merck (Darmstadt, Germany). Coupling reagent O-(7-azabenzotriazole-1-yl)-N,N,N′,N′-tetramethyluronium hexafluorophosphate (HATU) was obtained from ChemPep (Wellington, USA); trifluoroacetic acid (TFA) was purchased from Iris Biotech (Marktredwitz, Germany). 

### 2.2. Preparation of Soy-Fermented Product

Fermented soy was obtained from Centrale del Latte di Vicenza, (CLV, Vicenza, Italy) as previously reported [13]. Briefly, soy drink was prepared from an emulsion of white hilum soybean (maturity group 0+) (Bio Slym, Viadana, MN, Italy) and inoculated with *Lactobacillus delbrueckii* subsp. *bulgaricus* and *Streptococcus thermophilus* (DuPont Danisco, Bologna, Italy) to obtain a dessert with creamy consistency. Commercial fermented soy samples were collected on the day of production and then subjected to in vitro simulated gastrointestinal digestion.

### 2.3. Simulated Gastrointestinal Digestion of Fermented Soy 

Simulated gastrointestinal digestion was performed using the optimized INFOGEST method as described by Minekus M et al. (2014) and Brodkorb A et al. (2019) [10,11]. Briefly, freshly prepared simulated fluids were used in order to mimic the physiological conditions of human digestion. CaCl_2_•2H_2_O was added to fluids before use. Digestion was carried out in three sequential phases. In the oral phase, 10 g of soy fermented product was mixed with 8 mL of simulated salivary fluid (SSF) (15.1 mM KCl, 3.7 mM KH_2_PO_4_, 13.6 mM NaHCO_3_, 0.15 mM MgCl_2_•6H_2_O and 0.06 mM (NH_4_)_2_CO_3_) and 1.5 mM CaCl_2_(H_2_O)_2_. The solution was brought to pH 7.0 with 1 M NaOH and water was added to obtain a final volume of 20 mL. The mixture was incubated at 37 °C for 2 min at 150 rpm in an orbital shaker. In the gastric phase, the oral bolus was mixed with 16 mL of simulated gastric fluid (SGF) (6.9 mM KCl, 0.9 mM KH_2_PO_4_, 25 mM NaHCO_3_, 47.2 mM NaCl, 0.1 mM MgCl_2_(H_2_O)_6_ and 0.5 mM (NH_4_)_2_CO_3_)) and 0.15 mM CaCl_2_•2H_2_O. Then, 1 mL of pepsin solution, dissolved in water, was added to the mixture to give a 1:20 enzyme to substrate (E:S) ratio. The pH was lowered to 2.5 with 6 M HCl and the volume was brought to 40 mL with water. After 90 min at 37 °C at 150 rpm, the gastric chyme was mixed with 17 mL of simulated intestinal fluid (SIF) (6.8 mM KCl, 0.8 mM KH_2_PO_4_, 85 mM NaHCO_3_, 38.4 mM NaCl and 0.33 mM MgCl_2_•6H_2_O) and 0.6 mM CaCl_2_•2H_2_O. Next, 10 mL of pancreatin stock solution dissolved in SIF fluid was added to samples to reach (E:S) 1:20. Then, the pH was brought to 7.0 with 1 M NaOH in a final volume of 80 mL. The mixture was incubated for 2 h at 37 °C at 150 rpm. Finally, enzymes were inactivated by heating the digested soy at 85 °C for 15 min in order to stop the digestive process. Samples were maintained at −20 °C until extraction of peptides. 

### 2.4. Purification of Digested Fraction 

Peptide-enriched fractions were obtained by solid-phase extraction with STRATA C18 E cartridge (Phenomenex, Torrance, CA, USA). In the first step, the column was activated with 50 mL of 100% acetonitrile (ACN) and washed with 125 mL of 0.1% TFA aqueous solution. Digested samples (50 mL) were loaded onto the column. Peptide fractions were obtained by a discontinuous gradient step of ACN by elution with 5%, 30% and 50% ACN solutions (50 mL for each elution step). Following this, 5–30% ACN fraction was collected, lyophilized (Freeze Drier, Edwards, Burgess Hill, UK) and stored at −20 °C until further analysis [10,17]. The 5-30% ACN peptide-enriched fraction was further purified with preparative reversed-phase high-performance liquid chromatography (RP-HPLC). The lyophilized fraction (40 mg) was dissolved in 2 mL of 0.1% TFA aqueous solution and loaded onto a SNAP KP-C18-HS 12 g column (particle size 50 µm, surface area 400 m2/g, pore volume 0.95 mL/g, 90 Å pore diameter; Biotage^®^, Uppsala, Sweden). After an isocratic step at 0% ACN for 10 min, the gradient was linearly increased from 0% to 40% in 24 min and, finally, to 100% in 5 min. Flow rate was set to 12 mL/min and fractions were collected every minute and then subjected to lyophilization. Absorbance was measured by UV detection at λ = 220 nm and λ = 280 nm.

### 2.5. Liquid Chromatography-Tandem Mass Spectrometry (LC-MS/MS) Analysis 

The lyophilized fraction of interest was dissolved in 0.1% formic acid (FA) water solution. Then, 1 µg of the obtained solution (corresponding to a volume of 2.5 µL) was subjected to LC-MS/MS analysis using an LTQ-Orbitrap XL mass spectrometer (Thermo Fisher Scientific, Waltham, MA, USA) connected online with a nano-HPLC Ultimate 3000 (Dionex–Thermo Fisher Scientific, Waltham, MA, USA). Briefly, the solution was loaded onto a 10-cm pico-frit chromatographic column (75-µm internal diameter, 15-µm tip, New Objective) packed in-house with C18 material (Aeris Peptide 3.6 µm XB-C18, Phenomenex, Torrance, USA) at a flow rate of 8 µL/min. Peptides were separated at a flow rate of 250 nL/min with a gradient of ACN/0.1% FA increasing linearly from 3% to 40% in 20 min. Source temperature was set at 200 °C and capillary voltage at 1.2 kV. A data-dependent acquisition (DDA) method was applied: the instrument performed a full scan at high resolution (60,000) in the Orbitrap followed by the MS/MS fragmentation on the ten most intense ions acquired with collision-induced dissociation (CID) in the linear trap. 

### 2.6. Database Search and Peptide Identification

Raw data files were analyzed with the Proteome Discoverer software (version 1.4, Thermo Fisher Scientific, Waltham, MA, USA) connected to the Mascot Search engine (version 2.2.4, Matrix Science, London, UK). Protein and peptide identifications were conducted against the *Glycine max* section of the UniProt database (version June 2018), using the following parameters: no enzyme, precursor tolerance 10 ppm and fragment tolerance 0.6 Da. Furthermore, oxidation of methionine was considered as a variable modification. The algorithm Percolator was used to assess the false-discovery rate (FDR) and results were filtered using an FDR < 0.01 both at the protein and peptide levels. Proteins were grouped into families on the basis of the principle of maximum parsimony.

### 2.7. Molecular Docking Analysis

Molecular docking was used to perform an in silico screening of the affinity of the identified peptides with the Kelch domain of Keap1. The protein–peptide model was generated using, in parallel, CABS-Dock and GalaxyPepDock, providing, as the template, the Kelch domain of Keap1 (PDB 2FLU, chain X) and the sequence of the peptides [18,19]. Both the predicted models were refined with HADDOCK and the interactions between peptides and Keap1 were analyzed with PISA [20,21,22]. The library of peptides was scored according to the PISA results.

### 2.8. Peptide Synthesis 

The choice of the peptides to be synthesized was based on the sequence alignment with the reference proteins identified from the previous LC-MS/MS analysis and followed by molecular docking analysis in order to identify the peptides that best interact with Keap-1. The thirteen chosen peptides are **D-10-R** (DEQIPSHPPR), **N-14-G** (NALEPDHRVESEGG) and **V-11-R** (VNPESQQGSPR) from glycinin G4; **S-10-S** (SLVNNDDRDS), **K-14-L** (KEQQQEQQQEEQPL), **I-11-N** (IGINAENNQRN) and **F-12-Q** (FSREEGQQQGEQ) from β-conglycinin α subunit 2; **F-12-G** (FVDAQPQQKEEG), **F-12-E** (FGREEGQQQGEE) and **H-14-E** (HEQKEEHEWHRKEE) from β-conglycinin α’ subunit; **M-13-E** (MRKPQQEEDDDDE) from glycinin G2; and **G-14-I** (GKHQQEEENEGGSI) and **Y-9-E** (YLAGNQEQE) from glycinin G1. All these peptides belonged to reference proteins of the proteome of *Glycine max* and resulted with the following #PSM values after sequence alignment: 1 for **D-10-R**, 1 for **N-14-G**, 3 for **V-11-R**, 3 for **S-10-S**, 1 for **K-14-L**, 1 for **I-11-N**, 2 for **F-12-Q**, 1 for **F-12-G**, 1 for **F-12-E**, 1 for **H-14-E**, 1 for **M-13-E**, 1 for **G-14-I** and 17 for **Y-9-E**. The peptides selected for the experimental analyses were synthesized by the solid-phase technique, employing a fully automated peptide synthesizer (Syro II, MultiSynTech Gmbh). The assembly of the peptide chain was done stepwise on Wang resin preloaded with the first N-α-Fmoc-protected amino acidic residue. The Fmoc standard strategy was utilized throughout the assembly of the polypeptide chain, with the coupling reagent HATU [23,24]. The side-chain-protected amino acid building blocks were Fmoc-Arg(Pbf)-OH, Fmoc-Asn(Trt)-OH, Fmoc-Asp(O*t*Bu)-OH, Fmoc-Gln(Trt)-OH, Fmoc-Glu(O*t*Bu)-OH, Fmoc-His(Trt)-OH, Fmoc-Lys(Boc)-OH, Fmoc-Ser(*t*Bu)-OH, Fmoc-Trp(Boc)-OH and Fmoc-Tyr(*t*Bu)-OH. The peptides were deprotected and then cleaved from the resin with a solution made of 88% (*v/v*) trifluoroacetic acid (TFA), 5% (*w/v*) phenol, 5% (*v/v*) H_2_O and 2% (*v/v*) triisopropylsylane. Cleavage was ensured by shaking the peptide solution at RT for 2.5 h before removing the resin by vacuum filtration. The assembled peptides were precipitated with cold diethyl ether and a pellet was obtained by centrifugation. The precipitates were washed twice with cold diethyl ether volumes. Crude peptides were subjected to purification by means of flash chromatography (SP1, Biotage, Uppsala, Sweden) on a Biotage SNAP Ultra C18 12 g cartridge packed with Biotage HP-Sphere C18 25-µm spherical silica. Molecular masses and purity of the synthesized peptides were assessed by mass spectrometry using a MALDI-TOF/TOF mass analyzer (ABI 4800, AB Sciex, Framingham, MA, USA). α-Cyano-4-hydroxycinnamic acid (10 mg/mL aqueous 70% (*v/v*) ACN/0.1% TFA) was used as a matrix. Spectra were acquired in positive reflectron mode with an accelerating voltage of 20 kV and a middle-level laser energy (3000 arbitrary units). Typically, each MS spectrum was acquired by averaging 1500 laser shots.

### 2.9. Antioxidant Activities In Vitro with ABTS Tests

To analyze the antioxidant activity of samples, the ABTS test was employed [25]. The ABTS test uses the radical molecule ABTS^•+^ derived from the reaction of 7 mM ABTS with 2.46 mM potassium persulfate for 18 h in the dark. Briefly, 0.1 mL of peptide solution (0.1 mg/mL) reacted with 0.1 mL 0.08 mM ABTS^•+^ and the decrease in absorbance was measured at 415 nm. The final concentration of the analyzed peptides was 0.05 mg/mL. Trolox C was used as a standard for the calibration curve for the ABTS assay and the results were reported as Trolox Equivalent Antioxidant Capacity (TEAC). 

### 2.10. Cellular Studies

#### 2.10.1. Caco-2 Cell Model

For cellular tests, Caco-2 cells (human colon-rectal adenocarcinoma, obtained from DISCOG University of Padova, Padova, Italy) were cultured in DMEM (Dulbecco’s Modified Eagle’s Medium) supplemented with 10% FBS at 37 °C in 5% CO_2_ atmosphere.

#### 2.10.2. MTT Assay 

Caco-2 cells (1 × 10^4^) were seeded in a 96-well plate and treated with peptide fractions (0.05 mg/mL) to evaluate cell viability [16,26]. After treatment, the medium was removed and 0.1 mL MTT solution (0.5 mg/mL) in PBS (1×) was added and incubated for 3 h in the dark at 37 °C and 5% CO_2_. MTT solution was discarded and 0.1 mL of isopropanol/DMSO (9:1) was added to stop the reaction. After 15 min at 37 °C in the dark, absorbance (Abs_595-690_) was estimated with a plate reader (TECAN Infinite^®^ M200 PRO). Finally, percentage of cell viability was calculated. When indicated, oxidative stress was induced with 200 µM *tert*-butyl hydroperoxide (TbOOH) for 18 h after treatment with the peptide fractions. 

#### 2.10.3. Estimation of ROS Level 

ROS levels in the Caco-2 cell line were assessed using 5-chloromethyl-2’,7’-dichlorohydrofluorescein diacetate (CM-H_2_DCFDA, Molecular Probes, Thermo Fisher Scientific, Waltham, MA, USA) [16,26]. Caco-2 cells (1 × 10^4^) were seeded in a 96-well plate and treated with soy fractions (0.05 mg/mL) for 24 h. Cells were rinsed with 0.1 mL PBS (1×) and 10 mM glucose. Then, 0.08 mL/well of 10 µM CM-H_2_DCFDA was added. After 20 min in the dark, cells were washed as previously indicated. Oxidative stress conditions were induced by 0.1 mL of 250 µM TbOOH. Increment in fluorescence was estimated with a plate reader at 485 nm (λ excitation) and 527 nm (λ emission) for 90 min.

#### 2.10.4. Caco-2 Cell Lysates

Caco-2 cells (5 × 10^5^) were seeded in a 6-well plate and treated with the selected peptides (0.05 mg/mL) for 24 or 48 h in the presence or absence of 350 µM TbOOH and 2.5 mM H_2_O_2_. Subsequently, PBS was used to rinse cells and 100 μL of lysis buffer (RIPA buffer modified) containing 150 mM NaCl, 1% Triton X 100, 0.1% SDS, 0.5% DOC, 1 mM NaF, 1 mM EDTA, 5 mM Tris/HCl (pH 7.4), 0.1 mM PMSF and a protease inhibitor cocktail (Complete, Roche^®^, Basel, Switzerland) was added to samples for 45 min at 4 °C. Cell lysates were centrifuged for 5 min at 11,600× *g* and the Lowry method was applied for determining protein content [27]. 

#### 2.10.5. TrxR Activity

Cell lysates (50 µg of proteins) were used for the evaluation of TrxR enzymatic activity with the methods described by Tonolo F et al. 2018 [26]. TrxR activity was evaluated in cell lysates by following absorbance at λ = 412 nm. The results were expressed as percentage of the total TrxR activity referred to mg of protein employed in the assay (50 µg). Enzymatic activity was estimated after treatment of cells with peptides (0.05 mg/mL, 24 or 48 h) and 350 µM TbOOH or 2.5 mM H2O2 for 48 h.

#### 2.10.6. Nuclear Fraction and Nrf2 Detection by Western Blot

Caco-2 cells, seeded in T25 flasks, were treated with peptides (0.05 mg/mL) for 24 h. Nuclear and cytosolic fractions were obtained as follow [15,16]. Cells were lysed on ice for 15 min with 100 μL of buffer containing 10 mM Hepes/Tris pH 7.9, 0.1 mM EGTA, 0.1 mM EDTA, 0.1 mM PMSF, 10 mM KCl, 1 mM NaF and a protease inhibitor cocktail. Subsequently, IGEPAL (5% final concentration) was added, samples were mixed for 15 s and centrifuged at 1,000× *g* for 10 min at 4 °C. The pellet (nuclear fraction) was dissolved in 20 mM Hepes/Tris (pH 7.9), 1 mM EGTA, 1 mM EDTA, 0.4 M NaCl in the presence of 0.1 mM PMSF, 1 mM NaF and a protease inhibitor cocktail and mixed for 10–15 s every 2 min. Samples were centrifuged at 20,000× *g* for 10 min at 4 °C. Nuclear proteins (30 µg) were analyzed with SDS-PAGE (10%) and subjected to Western blot analysis in order to detect the level of Nrf2 protein; PCNA was used as standard. Nine Alliance software (Mini 9 17.01 version, Uvitec Alliance, Cambridge, UK) was applied for densitometry evaluation. 

#### 2.10.7. Quantification of Phase II Antioxidant Enzymes 

Cell protein lysates (30 µg) were subjected to SDS-PAGE (12%) and then Western blot analysis was performed with the enhanced chemiluminescence system and UVITEC (Alliance Q9 Advanced, Cambridge, UK) equipment using Nine Alliance software for densitometry quantification. The enzymes analyzed were superoxide dismutase 1 (SOD1), thioredoxin reductase 1 (TrxR1), glutathione reductase (GR) and NAD(P)H quinone oxydoreductase 1 (NQO1). GAPDH was used as a loading control.

#### 2.10.8. Lipid Peroxidation Assay 

Lipid peroxidation was tested with a modified protocol as described by Tonolo F. et al. 2020 [15,25]. Caco-2 cells (5 × 10^5^) were seeded in a 6-well plate in the presence of the peptides (0.05 mg/mL). After 24 h, 250 μM *tert*-butyl hydroperoxide (TbOOH) was added for 1 h and 30 min in order to induce oxidative stress. Then, cells were washed with 1 mL of PBS (1×) and subsequently treated with 1 mL of 0.1 N H_2_SO_4_ and 150 μL of 10% phosphotungstic acid. Samples were centrifuged at 15,600× *g* for 10 min and the treatment was repeated twice. Then, the dry pellets were dissolved in 0.35% IGEPAL, 0.014% BHT and 0.23% thiobarbituric acid in H_2_O/acetic acid (1:1) at the final volume of 0.25 mL and incubated at 95 °C for 60 min. Then, cooled samples were centrifuged at 15,600× *g* for 10 min. The supernatants were treated with 400 μL of n-butanol, vigorously mixed and centrifuged again for 15 min. Thiobarbituric acid reactive substances (TBARS) adduct was present in the upper phase and measured fluorometrically at 530 nm (Ex) and 590 nm (Em) using a TECAN plate reader.

#### 2.10.9. Simulation of Intestinal Absorption 

Caco-2 cells (8 × 10^4^) were seeded on Transwell^®^ inserts supports (0.4-µm pore sizes, 12-mm Ø, 1.12-cm^2^ grown surface; Corning Life Sciences, Tewksbury, MA, USA) [26]. Supports were placed in a 12 wells plate in order to create apical and basolateral compartments. For differentiation of cells, 21 days were required. To assess the formation and the integrity of the cell monolayer, transepithelial electrical resistance (TEER) was measured with a Millicell^®^ ERS2 volt ohmmeter (EDM Millipore, Darmstadt, Germany) and only monolayers having TEER higher than 1100 Ω x cm^2^ were used [26,28]. After 21 days, with an intact epithelial barrier, the analysis of intestinal absorption was carried out. Cell monolayer was washed three times with Hank’s balanced salt solution (HBSS) and 10 mM glucose and the plate was equilibrated at 37 °C for 30 min. Enriched peptide fractions (150 μg) or peptides (75 μg) were added in the apical compartment that contains 0.77 mL of HBSS. Apical and basolateral solutions were collected after 10 and 120 min at 37 °C and centrifuged at 11,600× *g* for 7 min, and the obtained supernatants were lyophilized. 

#### 2.10.10. Reversed-Phase High-Performance Liquid Chromatography (RP-HPLC) Analysis

The RP-HPLC analysis of the fractions recovered from both the apical and basolateral compartments were performed on a Waters 2695 Separation Module (Milfold, MA, USA) with a Waters 996 Photodiode Array Detector. Samples were eluted employing an Onyx Monolitich C18 100 mm × 4.6 mm LC column (Phenomenex, Torrance, CA, USA) with a linear gradient (0–60%) of ACN, 0.08% (*v/v*) TFA over 20 min at a flow rate of 2 mL/min. The column effluent was monitored by UV signal detection (λ = 220 nm) and the peaks were collected. Peak integration was performed with the HPLC offline software Millenium 32 (Waters, Milfold, MA, USA).

#### 2.10.11. MS Analysis

Analysis of the obtained fractions was performed on a MALDI-TOF/TOF 4800 mass spectrometer (AB Sciex, Framingham, MA, USA). External mass calibration was ensured by mass peptide standards (Sigma-Aldrich, St. Louis, MO, USA). A full MS scan was acquired using a range of m/z between 500 and 5000. On the basis of the abundance of the observed ions, further MS spectra were acquired by selecting a more specific m/z range. For the basolateral fractions, the lyophilized samples were dissolved in 50 µL of aqueous 25 % (*v/v*) ACN, 0.08% TFA. MS analysis was performed on 2 µL of these solutions after mixing them with 2 µL of α-cyano-4-hydroxycynnamic acid as matrix (10 mg/mL aqueous 70% (*v/v*) ACN/0.1% TFA). Samples were spotted on a stainless steel 386-well MALDI plate. Mass spectra were acquired in the positive ion reflector mode, with the following analytical conditions: laser intensity variable in the range 3000–3800 (arbitrary units), shots/sub-spectrum 50, total shots/spectrum 1500, accelerating voltage 20 kV. Data Explorer software (AB Sciex, Framingham, MA, USA) was employed for data analysis. 

#### 2.10.12. Statistical Analysis

Values are indicated as mean ± SD of at least three independent experiments. The analysis of variance was performed with the Tukey–Kramer multiple comparison test. InStat 3 (GraphPad Software, San Diego, CA, USA) software was utilized. Only differences with *p* < 0.05 were considered significant.

## 3. Results

Soy products (10 g) fermented with *Lactobacillus delbrueckii* subsp. *bulgaricus* and *Streptococcus thermophilus* were digested, as described in the Materials and Methods section, with pepsin and pancreatin in order to simulate gastro-intestinal digestion and enhance the release of bioactive peptides. The obtained samples were subjected to solid-phase extraction in order to achieve a peptide-enriched fraction (5–30% ACN). Subsequently, the latter was further purified through RP-HPLC. The obtained sub-fractions were tested in vitro and in a cellular model. Then, peptides present in the most antioxidant fractions were identified by LC-MS/MS analysis and database search.

### 3.1. Purification of Enriched Peptide Fractions

**Figure 1:**
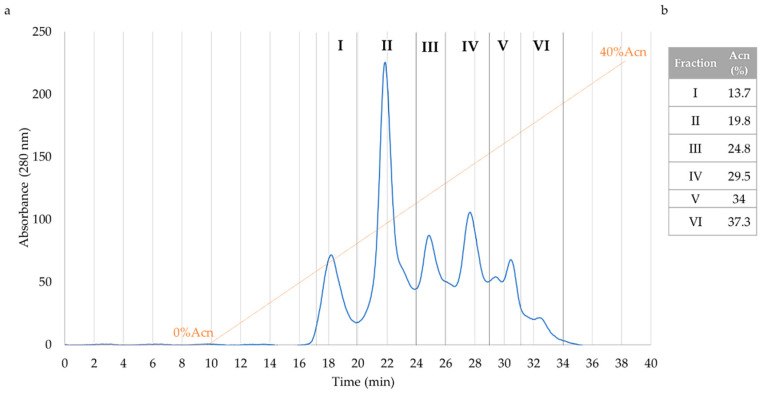
Purification of peptide fractions by reversed-phase high-performance liquid chromatography (RP-HPLC) analysis of 5–30% ACN digested soy fraction. (**a**) The analysis was performed with a linear gradient of 0–40% ACN over 20 min (12 mL/min flow rate). Fractions were collected on the basis of their absorbance at λ = 280 nm. (**b**) Percentage of ACN elution of each fraction.

First of all, simulated gastro-intestinal digestion on fermented soybean products (10 g) was performed in order to obtain potential bioactive peptides. From the obtained samples, employing solid-phase extraction, a peptide-enriched fraction (5–30% ACN) was obtained. The latter was tested in vitro and in a cellular model using Caco-2 cells. As reported in Table S1, 5–30% ACN showed high antioxidant activity in vitro and a significant protection of cell viability toward oxidative stress. In order to further separate the peptides extracted in the 5–30% ACN sample, RP-HPLC analysis was carried out, and six fractions were obtained, as indicated in Figure 1. In the further experimentation, the first five peptide-enriched fractions were considered, as in the last one, after the lyophilization step, the amount of the peptides was insufficient to perform any experiment, suggesting that a low amount of peptides was eluted. The main peak was eluted at 19.8% ACN, corresponding to sub-fraction II. In Figure 1b, percentage of ACN elution of peptides was reported for every purified fraction. In Figure S1, MS spectra of purified sub-fractions are presented. 

#### 3.1.1. Antioxidant Activity of Peptide-Enriched Fractions In Vitro and in Caco-2 Cells

ACN 5–30% and its purified sub-fractions (I, II, III, IV, V) were evaluated in vitro and in Caco-2 cells in order to estimate their antioxidant capacity. For the ABTS assay, peptide fractions (0.05 mg/mL) were tested with the free radical ABTS^+•^ as indicated in the Materials and Methods section. In Table S2, the results of the ABTS scavenging test carried out with the peptides are reported. In particular, sub-fractions IV and V showed a large antioxidant capacity in vitro.

**Figure 2:**
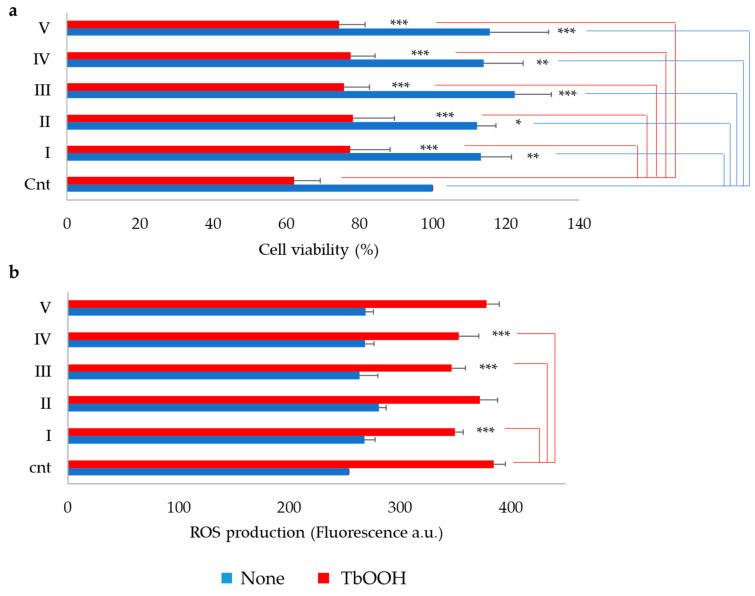
Cell viability and antioxidant activity of peptide fractions in Caco-2 cells. Cells were treated with peptide fractions (0.05 mg/mL) for 24 h. (**a**) The 3-(4,5-dimethylthiazolyl-2)-2,5-diphenyl tetrazolium bromide (MTT) test was carried out in the absence (blue bars) and in the presence (red bars) of 250 µM TbOOH (see Materials and Methods) to evaluate cell viability, and values re expressed as percentage of cell viability with respect to control. (**b**) CM-H_2_DCFDA was utilized for monitoring ROS production and the reported fluorescence was measured at 90 min (a.u.) in the absence (blue bars) or in presence (red bars) of 200 µM TbOOH. Values are indicated as mean ± SD of eight replicates of three independent experiments. *** *p* < 0.001, ** *p* < 0.01, * *p* < 0.05.

Cell viability and ROS production were evaluated in cells treated for 24 h with the purified sub-fractions (Figure 2). As a result, the fractions were not cytotoxic (Figure 2a). Furthermore, sub-fractions I, II, III, IV and V were able to significantly protect the cells toward oxidative stress induced by 200 μM TbOOH. To confirm the antioxidant activity observed in each fraction, ROS production was estimated in Caco-2 cells pre-treated with the peptide fractions (0.05 mg/mL) for 24 h, and then, oxidative stress was induced by 250 µM TbOOH, as described in the Materials and Methods section. This analysis revealed that cells treated with sub-fractions I, III and IV significantly decreased ROS production after oxidative stress induced by TbOOH with respect to cells treated only with TbOOH (Figure 2b).

#### 3.1.2. Simulation of Intestinal Absorption of Peptides 

In order to simulate the physiological intestinal absorption of the purified fractions, Caco-2 cells were seeded on Transwell^®^ inserts and, after 21 days of differentiation, treated with peptide-enriched fraction and sub-fractions (5–30%, I, II, III, IV, V). As reported in Figure S2, some peptides present in sub-fraction I were able to cross the simulated intestinal barrier after 120 min, showing no modifications of the peptides nor any formation of breakdown fragments (Figure S2 c,d; data for the other fractions are not reported). Sub-fraction I has been taken into consideration for further analysis because it showed the best results in terms of Caco-2 monolayer-crossing capacity with respect to the other fractions and proved to be highly protective against oxidative stress in the cell model by both MTT and ROS analyses. 

### 3.2. Identification of Bioactive Peptides in the Purified Fraction

The sequences of the peptides of the selected sub-fraction I were identified by nano-liquid chromatography/tandem mass spectrometry as detailed in the Materials and Methods section. A number of peptides from *Glycine max* proteins were confidently identified (Table S3). These derived mainly from several forms of soy β-conglycinin and glycinin. To reduce the number of possible candidates with antioxidant activity, peptides were firstly discriminated on the basis of their lengths (less than 20 amino acids) and masses to focus on those that can potentially translocate through the simulated intestinal barrier. A subset of these is reported in Table 1. The identified selected peptides were as follows: I-14-E from Glycinin G1; **Y-9-E** from Glycinin G1 and Glycinin G2 (i.e., this peptide sequence can be found in both native proteins); **R-11-D**, **Y-11-G**, **R-14-A** and **M-13-E** from Glycinin G2; and **N-9-R**, **D-10-R**, **V-11-R**, **W-11-E**, **N-14-G** and **K-16-V** from Glycinin G4. **S-10-S**, **V-10-Q**, **I-11-N**, **V-11-Q**, **F-12-Q**, **K-14-L** and **Q-13-K** were derived from β-conglycinin α subunit 2, while **T-10-S** and **F-12-G** originated from both β-conglycinin α’ subunit 2 and β subunit 2 (i.e., this peptide sequence can be found in both native proteins). **F-12-E** and **H-14-E** were derived from β-conglycinin α’ subunit and **Q-8-T** and **L-10-P** from β-conglycinin β-subunit 2. **N-12-S** and **F-10-V** were derived from Lectin. 

### 3.3. Molecular Docking Analysis

A molecular docking approach was used to select peptides with antioxidant properties. In particular, we tested their interaction in silico with the Keap1/Nrf2 system, the major signaling pathway involved in antioxidant capacity. In our previous works, some peptides identified from fermented milk showed antioxidant capacity through the activation of this pathway [15,16]. In this system, Nrf2 migrates to the nucleus, enhancing the cell defense response by stimulating the expression of antioxidant enzymes. The in silico model can predict the interaction between the Kelch domain of Keap1 with peptides of interest and allows for assigning an interaction-related score to each selected peptide, as indicated in Table 2. This score was evaluated considering the calculated dissociation energy of the system, the type and consistency of amino acidic interaction and the overall quality of the predicted complex. According to this scoring system, the peptides were sorted with a color code from green to red according to high to low probability of interaction, respectively. Peptides that showed more probability of interaction with Keap1 were selected (**D-10-R**, **N-14-G**, **S-10-S**, **K-14-L**, **F-12-G**, **V-11-R**, **I-11-N**, **F-12-E**, **H-14-E**, **M-13-E** and **G-14-I**) in order to evaluate their antioxidant activity in vitro and in the cellular model. In addition to the peptides identified having a high score, two other peptides with medium/low scores (**Y-9-E** and **F-12-Q**) were utilized as controls in subsequent testing. In Table S4, the predicted interaction of the selected peptides with Keap1 pocket is reported.

After this molecular docking analysis, the thirteen peptides reported were synthesized as described in Materials and Methods and employed for further investigations.

### 3.4. Antioxidant Capacity of Soy Peptides In Vitro and in Caco-2 Cell

An ABTS assay was carried out in order to test antioxidant activity of the selected peptides in vitro, as reported in Table S5. Values were indicated as TEAC (µM Trolox). **N-14-G**, **K-14-L**, **H-14-E**, **M-13-E** and **G-14-I** exhibited weak antioxidant capacity, while the other peptides were ineffective. 

**Figure 3:**
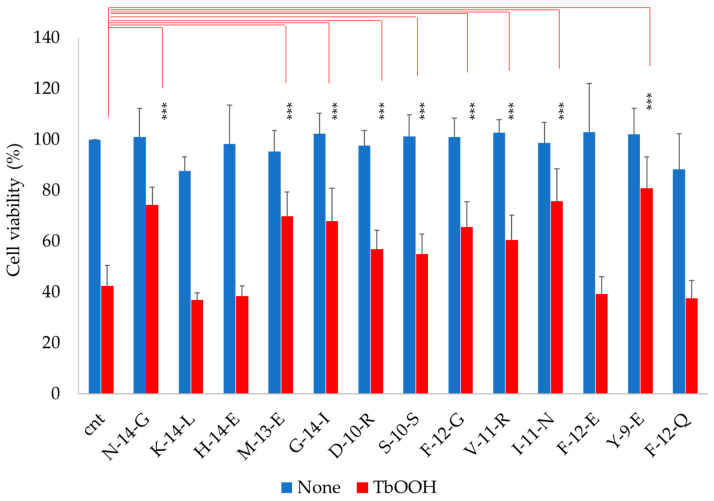
Analysis of cytotoxicity and antioxidant capacity in Caco-2 cells. The MTT test was performed in cells treated with soy peptides (0.05 mg/mL). Percentage of cell viability was observed after 24 h, while antioxidant capacity (as recovery of cell viability in oxidative stress conditions) was determined with 200 µM TbOOH for 18 h. Values are indicated as mean ± SD of eight replicates of three independent experiments. *** *p* < 0.001.

However, in the cellular model, the results were different with respect to in vitro analysis. In fact, the peptides were selected as described above, essentially for their capacity to inhibit the interaction of Keap1 with Nrf2 in cells. Caco-2 cells were utilized to investigate the antioxidant properties of the peptides. In more detail, Caco-2 cells were treated with peptides (0.05 mg/mL) for 24 h and subjected to MTT testing. As reported in Figure 3 (blue bars), the analyzed peptides were not cytotoxic compared to the viability of the control. In addition, the cells treated with the indicated peptides, showed a strong protection against oxidative stress induced by TbOOH. Indeed, when cells pre-treated for 48 h with the peptides were subjected to oxidative stress in the presence of 200 µM TbOOH for 18 h, some of these were able to restore viability with respect to the control in the presence of TbOOH (Figure 3, red bars). In particular, **N-14-G**, **M-13-E**, **G-14-I**, **D-10-R**, **S-10-S**, **F-12-G**, **V-11-R**, **I-11-N** and **Y-9-E**, after induction of oxidative stress, were able to significantly increase cell viability with respect to cells treated only with TbOOH. For example, a great rescue of the viability in cells treated with **N-14-G**, **V-11-R** and **I-11-N**, going from 42% in cells treated with only the oxidative agent to 74.3%, 60.3% and 75.6% for the three indicated peptides, respectively, was observed (Figure 3).

**Figure 4:**
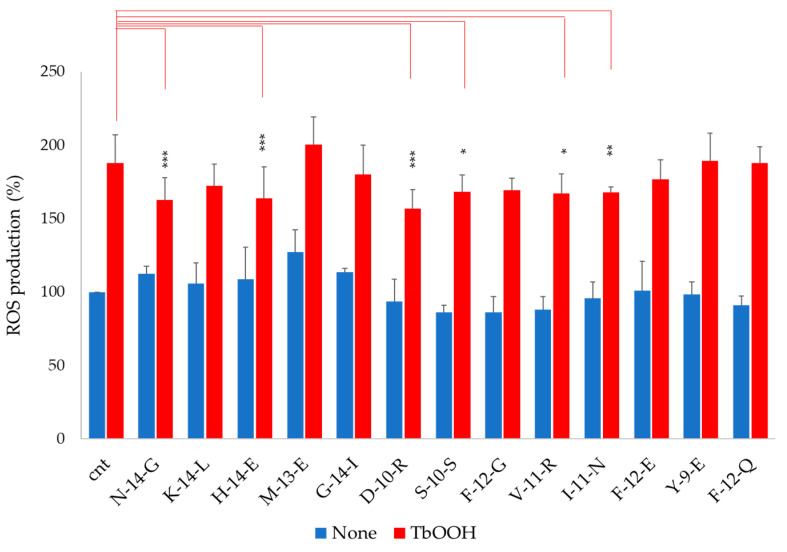
ROS production in Caco-2 cells treated with peptides. A CM-DCFDA probe was used and its fluorescence (a.u.) was measured in cells pre-treated with soy peptides alone (blue bars) or in the presence of 250 µM TbOOH (red bars). The reported values refer to fluorescence detected at 90 min. Values are indicated as mean ± SD of eight replicates of three independent experiments. *** *p* < 0.001, ** *p* < 0.01, * *p* < 0.05.

In order to confirm the antioxidant activity of peptides, ROS production in Caco-2 cells was estimated with the CM-DCFDA probe (Figure 4). As reported for the MTT test, cells were pre-treated with the indicated peptides for 24 h and then incubated for 20 min in the presence of the probe. The reaction started with the addition of 250 µM TbOOH. As reported in Figure 4, without the oxidative stimulus, no significant increase in ROS was observed in the presence of the peptides (blue bars). Furthermore, in the presence of TbOOH, in the cells pre-treated with **N-14-G**, **H-14-E**, **D-10-R**, **S-10-S**, **V-11-R** and **I-11-N**, ROS production was significantly lower than cells treated only with TbOOH (Figure 4, red bars). 

### 3.5. Mechanism of Action of Antioxidant Peptides

Due to the antioxidant effects that were exerted on Caco-2 cells, **N-14-G**, **D-10-R**, **S-10-S**, **V-11-R** and **I-11-N** were selected for further experiments and tested in order to elucidate their molecular mechanisms of action. The results obtained with other less-effective peptides are reported in the Supplementary Materials.

#### 3.5.1. Keap1/Nrf2 Pathway Activation 

**Figure 5:**
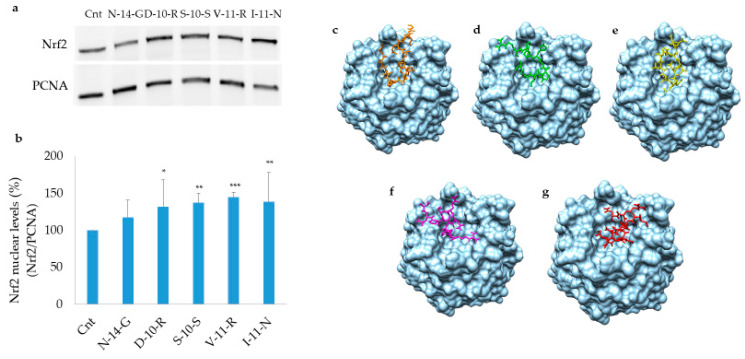
Nuclear factor erythroid 2-related factor 2 (Nrf2) translocation from cytosol to the nucleus in Caco-2 cells treated with peptides. (**a**) Nuclear fractions of cells treated with peptides were extracted and Western blot analysis was carried out to estimate Nrf2 levels. (**b**) Quantitative analysis of the reported Western blot after normalization using PCNA as a nuclear loading control. Values are indicated as mean ± SD of three independent experiments. (**c**–**g**) Binding geometry of **N-14-G** (**c**), **D-10-R** (**d**), **S-10-S** (**e**), **V-11-R** (**f**) and **I-11-N** (**g**) in the Keap1 Kelch domain pocket. *** *p* < 0.001, ** *p* < 0.01, * *p* < 0.05.

In order to define the molecular mechanism of action by which soy-derived bioactive peptides determine an antioxidant response in human cells, nuclear fractions were extracted from Caco-2 cells after treatment with **N-14-G**, **D-10-R**, **S-10-S**, **V-11-R** and **I-11-N** (0.05 mg/mL). Nrf2 levels were determined by Western blot analysis in the nuclear fraction (50 µg) to assess the activation of the Keap1/Nrf2 pathway. The selected peptides, in particular **D-10-R**, **S-10-S**, **V-11-R** and **I-11-N**, showed an increase in Nrf2 levels in the nucleus with respect to the control, while **N-14-G** was scarcely able to activate Nrf2 (Figure 5). Furthermore, **H-14-E** and **G-14-I** did not exhibit any stimulation of the Nrf2/Keap1 pathway (data not shown). The binding interaction of the peptides with Keap1 molecule is apparent in Figure 5, panels c–g.

#### 3.5.2. Overexpression of ARE-Regulated Enzymes 

**Figure 6:**
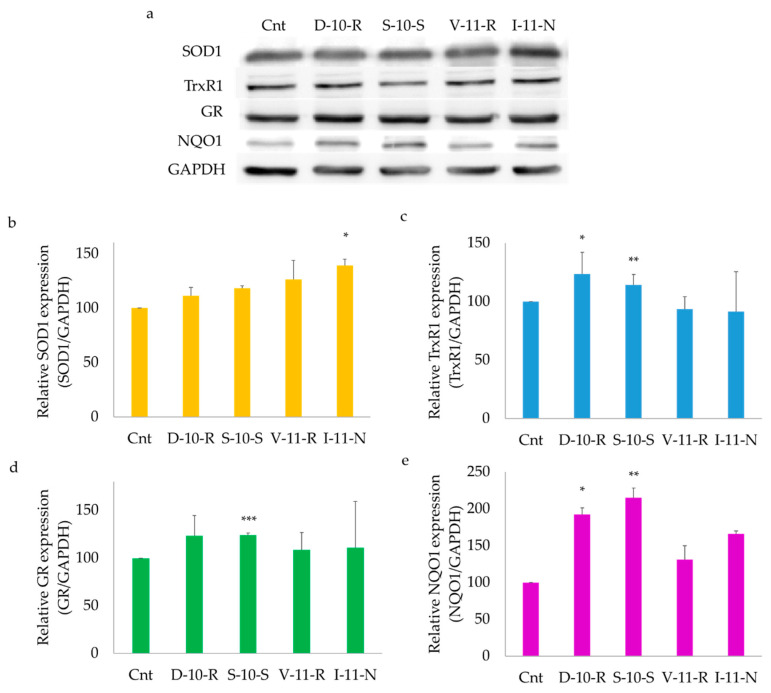
Expression of antioxidant and phase II enzymes. (**a**) Western blot analysis of antioxidant response element (ARE)-regulated enzymes. (**b**–**e**) Relative expression of superoxide dismutase 1 (SOD1), thioredoxin reductase 1 (TrxR1), glutathione reductase (GR) and NADPH Quinone oxidoreductase 1 (NQO1). GAPDH was used as a loading control to normalize the Western blot densitometric analysis. Values are indicated as mean ± SD of three independent experiments. *** *p* < 0.001, ** *p* < 0.01, * *p* < 0.05.

The peptides analyzed for activation of the Keap1/Nrf2 pathway were also tested in order to determine the expression of antioxidant and phase II enzymes, such as superoxide dismutase 1 (SOD1), thioredoxin reductase 1 (TrxR1), glutathione reductase (GR) and NADPH Quinone oxidoreductase 1 (NQO1). Western blot analysis was carried out in order to assess the expression of these antioxidant enzymes after induction of the Keap1/Nrf2 pathway. In Figure 6, the results obtained with **D-10-R**, **S-10-S**, **V-11-R** and **I-11-N** are reported, while in Figure S3, the effect of **N-14-G**, **K-14-L**, **M-13-E**, **H-14-E** and **G-14-I** on the expression of ARE-regulated enzymes is shown. With respect to the control cells, the peptides were able to determine an overexpression of the investigated enzymes and, in particular, **S-10-S** and **D-10-R** significantly increased the amount of the enzymes NQO1, GR and TrxR1, while **I-11-N** was effective at increasing the level of SOD1. 

#### 3.5.3. Total TrxR Enzymatic Activity

**Figure 7:**
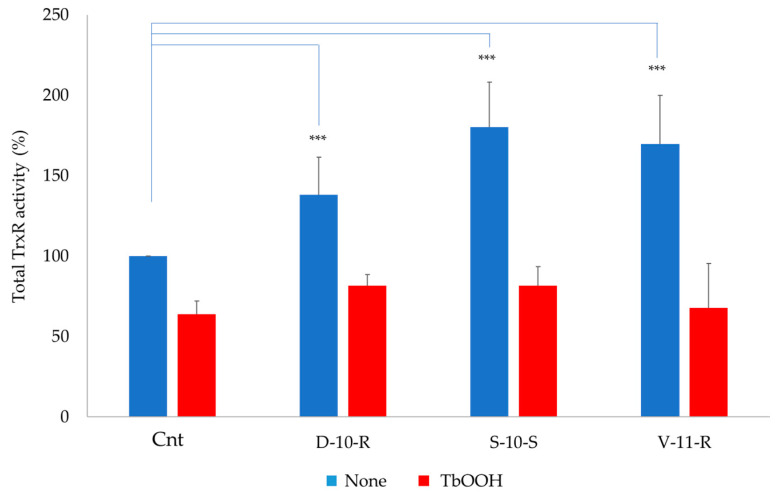
TrxR activity in Caco-2 cell lysates treated with peptides (0.05 mg/mL) in the presence or absence of TbOOH (350 µM). Cells were pre-treated with the peptides and oxidative stress was induced by 350 µM TbOOH for 48 h. Total TrxR activity was measured at 412 nm and referred to mg proteins. Values are indicated as mean ± SD of 2 replicates of 3 independent experiments. *** *p* < 0.001.

Total activity of TrxR was analyzed in Caco-2 cells treated for 24 (Figure S4) and 48 h (Figure 7 and Figure S6) with the selected peptides, in particular with **D-10-R**, **S-10-S** and **V-11-R**. The presence of the peptides, in particular **D-10-R**, in cells incubated for 24 h induced a TrxR activity higher than the control (Figure S4). Moreover, the peptides, in particular **D-10-R** and **S-10-S**, can slightly rescue the activity of TrxR after oxidative stress induced by 350 µM TbOOH for 48 h (Figure 7). In addition, as reported in Figure S5, in cells treated for 48 h with 2.5 mM H_2_O_2_, the activity of the analyzed antioxidant enzymes increased as a consequence of activation of the Keap1/Nrf2 pathway. At the same time, when cells were pre-treated with **D-10-R**, **S-10-S** and **V-11-R** and oxidative stress was induced by 2.5 mM H_2_O_2_, the activity was higher than the control treated only with H_2_O_2_. This result showed that there was an additive effect on the activation of the antioxidant pathway. 

#### 3.5.4. Lipid Peroxidation Assay

**Figure 8:**
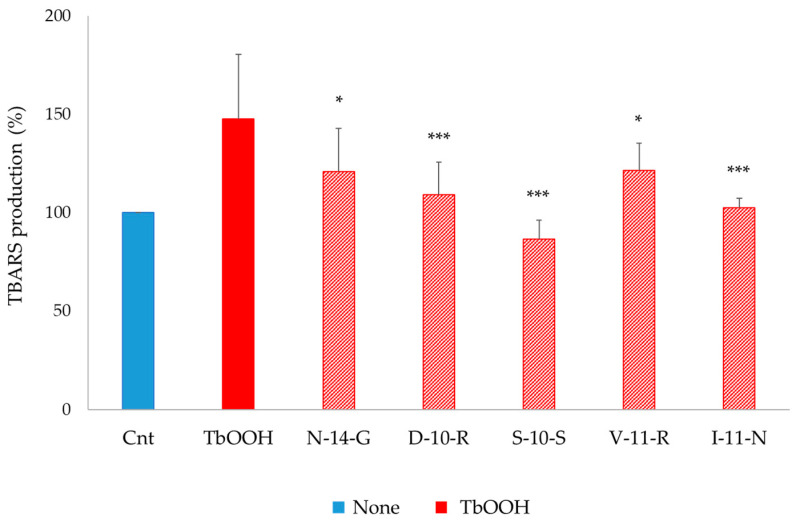
TBARS adducts production (%) in Caco-2 cells. Caco-2 cells (5 × 10^5^) were pre-treated with the peptides (0.05 mg/mL) for 24 h and oxidative stress was induced with 250 µM TbOOH for 1 h and 30 min. Lipid peroxidation was estimated fluorometrically. Values are indicated as mean ± SD of two replicates of three independent experiments. *** *p* < 0.001, * *p* < 0.05.

**N-14-G**, **D-10-R**, **S-10-S**, **V-11-R** and **I-11-N** peptides were tested for lipid peroxidation estimated as TBARS production, as described in the Materials and Methods section. Caco-2 cells were pre-treated with the peptides, and after 24 h, 250 µM TbOOH was added in order to induce oxidative cell damage. As reported in Figure 8, all the selected peptides were able to protect from the oxidation exerted by TbOOH. In particular, **S-10-S** was able to completely prevent lipid peroxidation, showing values similar to the untreated control.

## 4. Discussion

Simulated gastro-intestinal digestion is a useful tool to obtain potential bioactive peptides deriving from fermented soybean products. This methodology was used in order to mimic, as much as possible, the conditions that occur physiologically in the human gastro-intestinal tract. The correct choice of the enzymes, pH and salt concentrations of the different phases and the time of the digestion procedure were established. The digested products were subjected to an initial step using solid-phase extraction obtaining the 5–30% ACN peptide-enriched fraction. The latter was tested for its antioxidant activity in our cellular model, showing a significant protection against oxidative stress induced by TbOOH, as demonstrated in Figure 2. The 5–30% ACN fraction was then subjected to further RP-HPLC purification using a continuous LC gradient, thus obtaining six sub-fractions on the basis of absorbance intensity at 280 nm as an index of peptide abundance in each fraction (Figure 1). Fractions I to V were tested in vitro to evaluate their antioxidant properties with the ABTS test and in Caco-2 cells in order to demonstrate their ability to simulate intestinal absorption and their antioxidant capacity. Fractions I to V were able to protect against oxidative stress, as shown by the MTT test and ROS analysis (Figure 2). However, among these five fractions, peptides contained in fraction I were particularly effective in crossing the simulated intestinal barrier, obtained by cells arranged in a monolayer, with more efficiency than the other fractions. Thus, further investigations were carried out to identify the peptides present in this fraction, which were subjected to LC-MS/MS analysis, and spectra were searched against the *Glycine max* section of the UniProt database, yielding a list of high-confidence peptide sequences (Table S3). After sequence alignment of the identified peptides with the reference proteins from *Glycine max* proteome, an initial selection of 28 peptides was made based on some criteria such as the length of the peptides, which should be less than 20 amino acids, and molecular mass lower than 2000 Da. Moreover, when more than one peptide covered a single region on the original protein, only the peptide with the shorter sequence was chosen. Following this first selection, peptide sequences were further shortlisted based on the results of a molecular docking approach, which allowed us to greatly reduce the number of peptides to be synthesized and tested in vitro. As shown in our previous works [15,16], peptides that interact with the Kelch domain of Keap1 activate the Keap1/Nrf2 pathway. In silico molecular docking can consistently predict this interaction and can also provide details of its nature. In this work, we show that a molecular docking approach can successfully be applied to perform an in-silico pre-screening and effectively identify peptides endowed with a high affinity to the target protein. This in silico pre-screening produced a scoring method based on the calculated dissociation energy of the system, the type and consistency of amino acidic interaction and the overall quality of the predicted complex. Therefore, it provides a first insight of peptides’ interaction before the in vitro analysis, allowing to focus on the peptides that show a more significant affinity. This approach was fundamental to evaluate in silico the activation of the Keap1/Nrf2 system by each peptide, as this is one of the most important signaling pathways in the cellular antioxidant response. From this screening strategy, a panel of thirteen peptides was selected (**D-10-R**, **N-14-G**, **S-10-S**, **K-14-L**, **F-12-G**, **V-11-R**, **I-11-N**, **F-12-E**, **H-14-E**, **M-13-E**, **G-14-I**, **Y-9-E** and **F-12-Q**). All of them were absent in the BIOPEP database, meaning that they were not previously investigated as bioactive peptides in the literature [30]. These thirteen peptides were then synthesized utilizing a solid-phase technique in an automated peptide synthesizer and they were then tested for their antioxidant properties, both in vitro and in a cellular model. 

The antioxidant test ABTS showed that **N-14-G**, **K-14-L**, **H-14-E**, **M-13-E** and **G-14-I** presented low antioxidant capacity *in vitro*, while the other peptides were ineffective. On the other side, in cells treated with TbOOH, **N-14-G**, **M-13-E**, **G-14-I**, **D-10-R**, **S-10-S**, **F-12-G**, **V-11-R**, **I-11-N** and **Y-9-E** promoted cell viability, which was significantly increased with respect to control cells. The antioxidant mechanism of action of the selected peptides was also investigated, at first by monitoring the activation of the Keap1/Nrf2 pathway, which was assessed through increase in Nrf2 levels in the nucleus. This increase confirmed that the selected peptides interacted with Keap1, allowing Nrf2 translocation to the nucleus and consequently activating the expression of the enzymes involved in protection against oxidative agents. The peptides that best mediated the increase of Nrf2 levels in the nucleus, determined by Western blot analysis, were **D-10-R**, **S-10-S**, **V-11-R** and **I-11-N** (Figure 5 and Figure S3). Then, the overexpression of ARE-controlled enzymes, such as superoxide dismutase 1 (SOD1), thioredoxin reductase 1 (TrxR1), glutathione reductase (GR) and NADPH-quinone oxidoreductase 1 (NQO1), was shown to be mediated by activation of the Keap1/Nrf2 pathway after peptide treatment in Caco-2 cells. This analysis revealed that the treatment with peptide **S-10-S** significantly increased the expression of these three enzymes. Furthermore, we evaluated the activity of TrxR1 in Caco-2 cells treated for 24 h with the synthesized peptides (Figure S4) and demonstrated that the **D-10-R** peptide is particularly effective in activating this enzyme. In addition to **D-10-R**, **S-10-S** can also rescue the TrxR1 activity after oxidative stress induced by TbOOH (Figure 7). Both these two peptides (**D-10-R** and **S-10-S**), together with **V-11-R**, when added to Caco-2 cells treated with H_2_O_2_, can increase the activity of the TrxR1 enzyme with respect to cells treated only with the oxidative agent (Figure S5). Finally, based on the results obtained by the previous analyses, five peptides (i.e., **N-14-G**, **D-10-R**, **S-10-S**, **V-11-R** and **I-11-N**) were selected to assess their effects on the levels of TBARS adduct after oxidative stress induced by TbOOH. While all tested peptides were able to protect cells toward lipid peroxidation, the best performing one (i.e., the **S-10-S** peptide) showed the capacity to reduce TBARS adducts production to the levels of the untreated cells (Figure 8).

## 5. Conclusions

Peptides derived from a fermented soy product subjected to simulated gastro-intestinal digestion mimicking the physiological conditions of the human body were identified, shortlisted based on their size and molecular weight and further screened with an innovative molecular docking approach to select those with the highest predicted affinity for Keap1. The peptides were synthesized and tested for their antioxidant properties both in vitro and in a cellular model. 

Fermented soy-derived bioactive peptides exhibited important antioxidant properties against oxidative stress, showing their positive effects both at the level of cell viability as well as ROS production. The antioxidant properties of the bioactive peptides were assessed by monitoring their ability to activate the Keap1/Nrf2 pathway, determining the translocation of Nrf2 from the cytosol to the nucleus, and eventually leading to the transcription of antioxidant genes.

In particular, **D-10-R** and **S-10-S** demonstrated high protective effects in various biochemical assays, with **S-10-S** being the best performing one also for its ability to inhibit lipid peroxidation. Those actions can essentially be attributable to activation of the Keap1/Nrf2 pathway, demonstrated by Nrf2 translocation from the cytosol to the nucleus, increased expression of ARE-regulated enzymes, induction of TrxR1 enzymatic activity and prevention of lipid peroxidation. 

Noteworthily, all these observations give a new insight on the antioxidant action. In particular, in the classic activation of the Keap1/Nrf2 pathway, oxidants or electrophilic molecules, such as isothiocyanates, induce transient modifications to Keap1 by interacting with specific Cys residues. Based on the collected evidence, we propose here a possible alternative molecular mechanism of action of antioxidant bioactive peptides, according to which these food-derived molecules can directly interact with the amino acidic residues of the pocket of Keap1 involved in the binding of the Neh domain of Nrf2. 

## Figures and Tables

**Table 1:** Selected peptides from Orbitrap analysis identified by liquid chromatography/tandem mass spectrometry (LC-MS/MS) analysis with a sequence length < 20 amino acids and a MW < 2000 Da.

Sequence	Peptide Name	Native Protein	MW (Da) Monoisotopic	Number aa	#PSM	Ion Score
YLAGNQEQE	**Y-9-E**	Glycinin G1–G2	1050.45	9	17	54
IKPPTDEQQQRPQE	**I-14-E**	Glycinin G1	1692.83	14	2	41
GKHQQEEENEGGSI	**G-14-I**	Glycinin G1	1540.66	14	1	23
RQNIGQNSSPD	**R-11-D**	Glycinin G2	1214.55	11	2	41
YQEPQESQQRG	**Y-11-G**	Glycinin G2	1348.59	11	1	55
RNLQGENEEEDSGA	**R-14-A**	Glycinin G2	1546.63	14	1	52
MRKPQQEEDDDDE	**M-13-E**	Glycinin G2	1633.64	13	1	41
NNQLDQTPR	**N-9-R**	Glycinin G4	1084.51	9	2	50
DEQIPSHPPR	**D-10-R**	Glycinin G4	1174.56	10	1	37
VNPESQQGSPR	**V-11-R**	Glycinin G4	1197.56	11	3	79
WQEQQDEDEDE	**W-11-E**	Glycinin G4	1449.50	11	1	44
NALEPDHRVESEGG	**N-14-G**	Glycinin G4	1508.67	14	1	49
KQGQHQQEEEEEGGSV	**K-16-V**	Glycinin G4	1797.76	16	2	51
SLVNNDDRDS	**S-10-S**	β-conglycinin α subunit 2	1133.48	10	3	48
VGLKEQQQEQ	**V-10-Q**	β-conglycinin α subunit 2	1185.58	10	1	44
IGINAENNQRN	**I-11-N**	β-conglycinin α subunit 2	1241.60	11	1	67
VGLKEQQQEQQ	**V-11-Q**	β-conglycinin α subunit 2	1313.64	11	1	37
FSREEGQQQGEQ	**F-12-Q**	β-conglycinin α subunit 2	1421.60	12	2	59
KEQQQEQQQEEQPL	**K-14-L**	β-conglycinin α subunit 2	1768.81	14	1	58
QREEQEWPRKEEK	**Q-13-K**	β-conglycinin α subunit 2	1770.85	13	1	25
TLVNNDDRDS	**T-10-S**	β-conglycinin α’ subunit 2/β subunit 2	1147.49	10	2	45
FVDAQPQQKEEG	**F-12-G**	β-conglycinin α’ subunit 2/β subunit 2	1374.63	12	1	48
FGREEGQQQGEE	**F-12-E**	β-conglycinin α ’subunit	1392.58	12	1	60
HEQKEEHEWHRKEE	**H-14-E**	β-conglycinin α ’subunit	1929.86	14	1	12
QRIPAGTT	**Q-8-T**	β-conglycinin β subunit 2	842.45	8	1	31
LKVREDENNP	**L-10-P**	β-conglycinin β subunit 2	1212.59	10	1	30
NKVDENGTPKPS	**N-12-S**	Lectin	1284.62	12	4	57
FNENESGDQV	**F-10-V**	Lectin	1137.44	10	1	61

Bold highlights the names of the peptides.

**Table 2:** Molecular docking analysis of soy peptides.

	Sequence	Peptide	MW (Da)	Class of Amino Acids (%)				pI (Theoretical)
				Basic	Acid	Neutral	Hydrophobic	
	DEQIPSHPPR	**D-10-R**	1174.56	20	20	50	10	5.32
	NALEPDHRVESEGG	**N-14-G**	1508.67	14.29	28.57	35.71	21.43	4.4
	SLVNNDDRDS	**S-10-S**	1133.48	10	30	40	20	3.93
	KEQQQEQQQEEQPL	**K-14-L**	1768.81	7.14	28.57	57.14	7.14	4.09
	FVDAQPQQKEEG	**F-12-G**	1374.63	8.33	25	41.67	25	4.14
	VNPESQQGSPR	**V-11-R**	1197.56	9.09	9.09	72.73	9.09	5.97
	IGINAENNQRN	**I-11-N**	1241.60	9.09	9.09	54.55	27.27	6
	FGREEGQQQGEE	**F-12-E**	1392.58	8.33	33.33	50	8.33	4.09
	HEQKEEHEWHRKEE	**H-14-E**	1929.86	42.86	42.86	7.14	7.14	5.39
	MRKPQQEEDDDDE	**M-13-E**	1633.64	15.38	53.85	23.08	7.69	3.9
	GKHQQEEENEGGSI	**G-14-I**	1540.66	14.29	28.57	50	7.14	4.48
	YLAGNQEQE	**Y-9-E**	1050.45	0	22.22	44.44	33.33	3.79
	YQEPQESQQRG	**Y-11-G**	1348.59	9.09	18.18	63.64	9.09	4.53
	RQNIGQNSSPD	**R-11-D**	1214.55	9.09	9.09	72.73	9.09	5.84
	NNQLDQTPR	**N-9-R**	1084.51	11.11	11.11	66.67	11.11	5.84
	KQGQHQQEEEEEGGSV	**K-16-V**	1797.76	12.5	31.25	50	6.25	4.32
	VGLKEQQQEQ	**V-10-Q**	1185.58	10	20	50	20	4.53
	VGLKEQQQEQQ	**V-11-Q**	1313.64	9.09	18.18	54.55	18.18	4.53
	QRIPAGTT	**Q-8-T**	842.45	12.5	0	62.5	25	9.75
	LKVREDENNP	**L-10-P**	1212.59	20	30	30	20	4.68
	NKVDENGTPKPS	**N-12-S**	1284.62	16.67	16.67	58.33	8.33	6.07
	FNENESGDQV	**F-10-V**	1137.44	0	30	50	20	3.57
	RNLQGENEEEDSGA	**R-14-A**	1546.63	7.14	35.71	42.86	14.29	3.91
	IKPPTDEQQQRPQE	**I-14-E**	1692.83	14.29	21.43	57.14	7.14	4.68
	FSREEGQQQGEQ	**F-12-Q**	1421.60	8.33	25	58.33	8.33	4.25
	WQEQQDEDEDE	**W-11-E**	1449.50	0	63.64	27.27	9.09	3.26
	TLVNNDDRDS	**T-10-S**	1147.49	10	30	40	20	3.93
	QREEQEWPRKEEK	**Q-13-K**	1770.85	30.77	38.46	23.08	7.69	5.07

Soy peptides were evaluated with molecular docking analysis for their interaction in silico with Keap1. A score was assigned to each selected peptide and the probability of interaction with Keap1 is represented here with a color scale, i.e., from lower (red) to higher (green). Characteristics of amino acids present in all peptides were determined by the Peptide Property Calculator database. Theoretical isoelectric point (pI) was calculated with ProtParam database [29]. Bold highlights the names of the peptides.

## Supplementary Materials

The following are available online at https://www.mdpi.com/2076-3921/9/12/1306/s1, Figure S1. Examples of mass spectrometry spectra of sub-fractions I, II, III, IV and V; Figure S2. Transepithelial transport in Caco-2 cell monolayer of sub-fraction I; Figure S3. Western blot analysis of antioxidant and phase II enzymes in Caco-2 cell lysates treated with **N-14-G**, **K-14-L**, **H-14-E**, **M-13-E** and **G-14-I** (0.05 mg/mL) for 24 h; Figure S4. GR and TrxR activity in Caco-2 cell lysates treated with soy peptides (0.05 mg/mL) for 24 h; Figure S5. Total TrxR activity (%) in Caco-2 cells treated with **D-10-R**, **S-10-S** and **V-11-R** (0.05 mg/mL) in the presence or absence of 2.5 mM H_2_O_2_ for 48 h; Table S1. Analysis of the antioxidant activity of 5–30% ACN in vitro and in a cellular model; Table S2. Analysis of the antioxidant activity of sub-fractions I, II, III, IV and V in vitro; Table S3. Files from Orbitrap analysis of sub-fraction I; Table S4. ABTS assay with identified soy peptides (0.05 mg/mL).
