# Supplementary material for: Fermented Soy-Derived Bioactive Peptides Selected by a Molecular Docking Approach Show Antioxidant Properties Involving the Keap1/Nrf2 Pathway
Source: Antioxidants (Basel). 2020 Dec 19;9(12):1306. doi: 10.3390/antiox9121306 (PMC7765838; doi:10.3390/antiox9121306)
Supplement: Supplementary file 1 [file antioxidants-09-01306-s001.pdf]

Fermented soy-derived bioactive peptides selected by a molecular docking approach show antioxidant properties involving the Keap1/Nrf2 pathway in a cellular model

Federica Tonolo [1,#], Laura Moretto [1,#], Alessandro Grinzato [1,#], Federico Fiorese [1], Alessandra Folda [1], Valeria Scalcon [1], Stefania Ferro [1], Giorgio Arrigoni [1,2,3], Marco Bellamio [4], Emiliano Feller [4], Alberto Bindoli [5], Oriano Marin [1,*], and Maria Pia Rigobello [1,*]

[1] Department of Biomedical Sciences, University of Padova, via Ugo Bassi 58/b, 35131 Padova, Italy
[2] Proteomics Center, University of Padova and Azienda Ospedaliera di Padova
[3] CRIBI, Biotechnology Center, University of Padova
[4] Centrale del Latte di Vicenza, UO di Centrale del Latte d'Italia, via Alessandro Faedo, 60 Vicenza;
[5] Institute of Neuroscience (CNR), viale G. Colombo 3, 35131, Padova, Italy;
* Correspondence: mariapia.rigobello@unipd.it;
[#] These authors contributed equally

Table S1. Analysis of the antioxidant activity of 5-30% ACN *in vitro* and in a cellular model. ABTS and DPPH assays were performed for *in vitro* analysis and values were indicated respectively as TEAC (μM Trolox C) and percentage of DPPH scavenging inhibition. MTT test was carried out in order to evaluate cell viability in Caco-2 cells treated with the enriched peptide fraction for 24 h. Oxidative stress was induced with TbOOH (200 μM) for 18 h. n.d.: not detectable; *** p < 0.001

	***In vitro* analysis**		**Cell viability in Caco-2 cells**	
	**TEAC (μM Trolox C)**	**DPPH scavenging inhibition (%)**	**None**	**TbOOH**
Cnt	n.d.	n.d.	100.00 ± 0.00	62.11 ± 7.19
5-30% ACN fraction	0.57 ± 0.37 (***)	15.27 ± 5.98 (***)	123.26 ± 8.44	95.06 ± 10.96 (***)

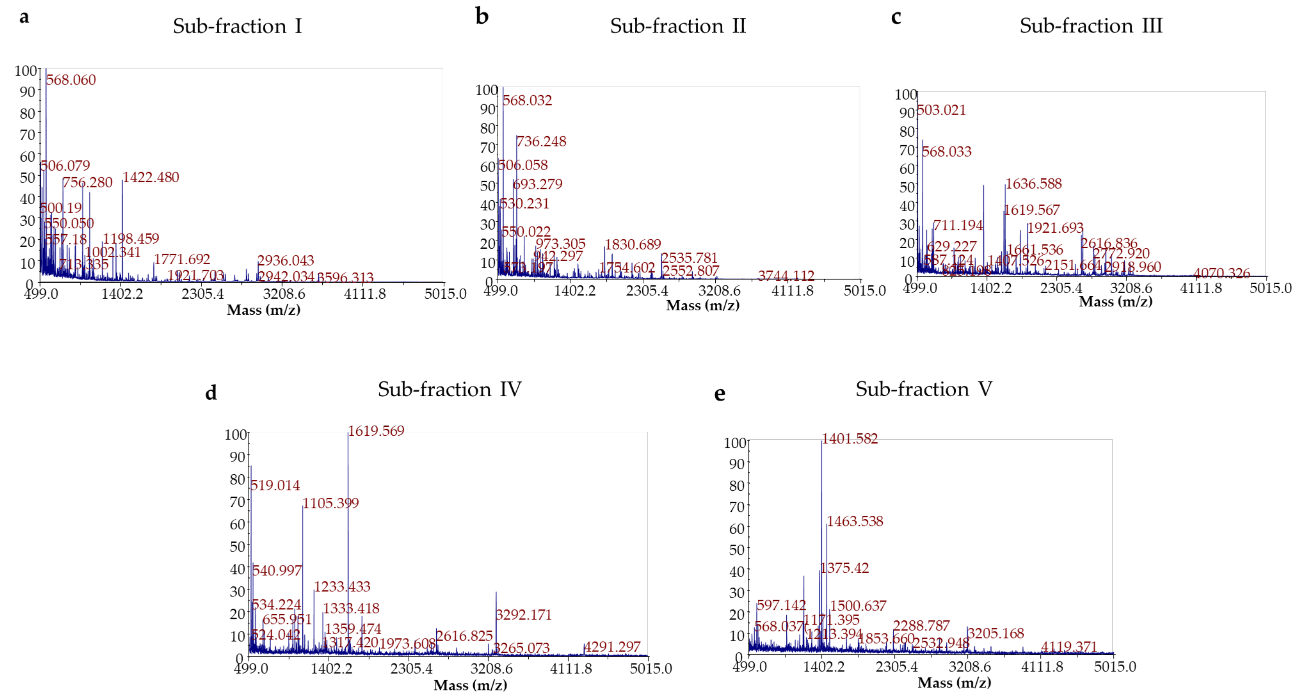

Figure S1. Examples of mass spectrometry spectra of sub-fractions I, II, III, IV and V. **a)** Sub-fraction I. **b)** Sub-fraction II. **c)** Sub-fraction III. **d)** Sub-fraction IV. **e)** Sub-fraction V.

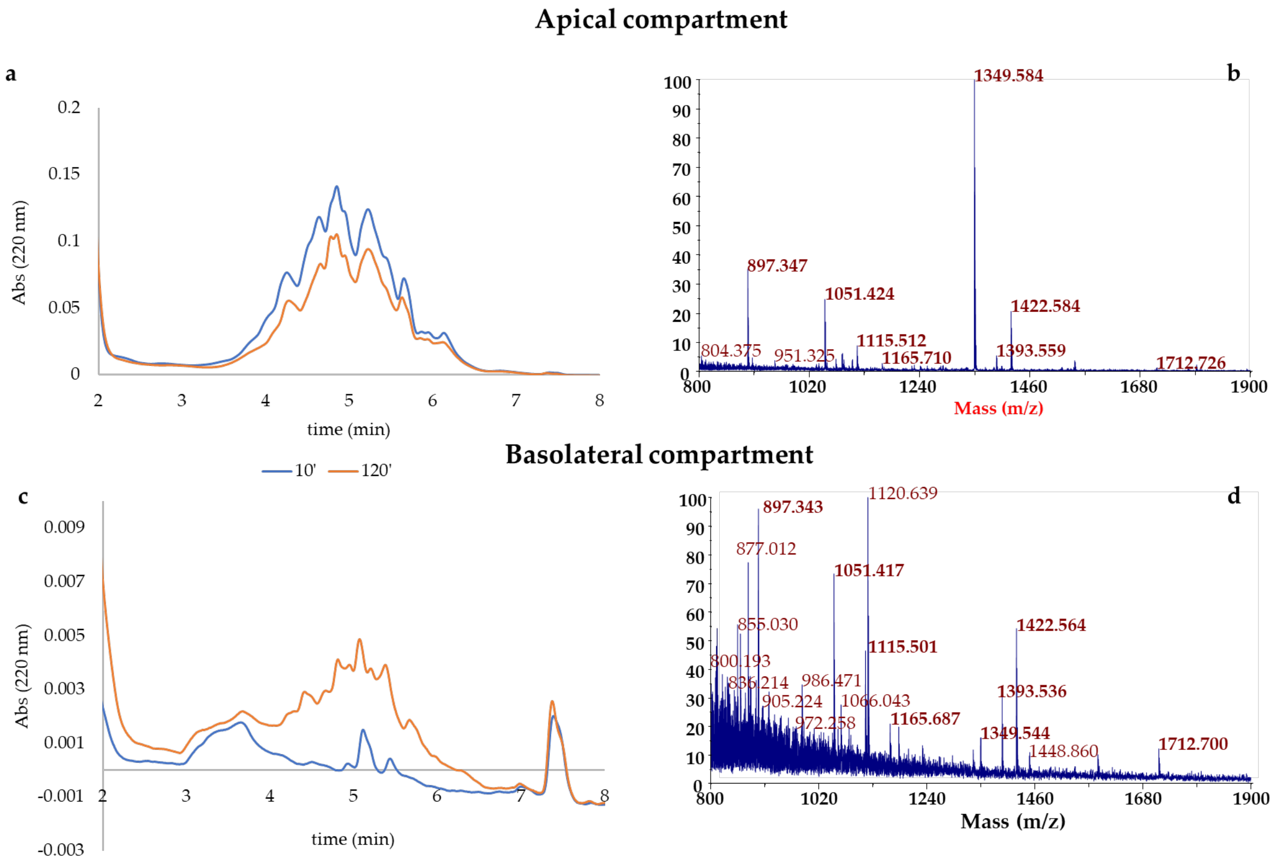

Figure S2. Transepithelial transport in Caco-2 cell monolayer of sub-fraction I. Cells were grown on Transwell inserts for 21 days to let their differentiation. Then the experiment was carried out as reported in Materials and Methods section. RP-HPLC analysis of apical (AP) compartment **(a)** and basolateral (BL) compartment **(c)** of cells treated with sub-fraction I for 10 min (blue) and 120 min (orange) are reported. Mass spectrometry analysis of AP compartment **(b)** and BL compartment **(d)** of cells treated with sub-fraction I for 120 min are depicted. Peptides that are able to cross the Caco-2 monolayer without modifications are reported in bold.

Table S2. Analysis of the antioxidant activity of sub-fractions I, II, III, IV and V *in vitro*. ABTS assay were performed for *in vitro* analysis and values were indicated respectively as TEAC (μM Trolox C). *** $p < 0.001$

Sub-fraction	TEAC (μM Trolox C)
**I**	0.95 ± 1.34
**II**	1.87 ± 1.15 (***)
**III**	1.95 ± 1.52 (***)
**IV**	2.64 ± 1.41 (***)
**V**	2.78 ± 1.18 (***)

Table S3. Files from Orbitrap analysis of sub-fraction I

	Sequence	# PSMs	# Proteins	# Protein Groups	Protein Group Accessions	Modifications	ΔCn	q-Value	PEP	IonScore	Exp Value	Charge	MH+ [Da]	ΔM [ppm]	RT [min]
**High**	VNPESQQGSPR	3	2	1	P02858		0.0000	0	0.00001754	79	3.29476E-05	2	1198.58220	0.88	13.23
**High**	REQDEDEDEDEDKPRPSRPSQG	2	2	1	P02858		0.0000	0	0.0000027	54	0.004150825	4	2614.12905	1.58	13.60
**High**	KQGQHQQEEEEEGGSV	2	2	1	P02858		0.0000	0	0.00003189	51	0.010716809	2	1798.78740	1.95	12.92
**High**	NNQLDQTPR	2	2	1	P02858		0.0000	0	0.0005204	50	0.019479559	2	1085.53459	1.05	14.78
**High**	NALEPDHRVESEGG	1	3	2	P02858; A0A0R0GMV1		0.0000	0	0.005443	49	0.031992697	2	1509.69512	1.47	15.69
**High**	EQDEDEDEDEDKPRPSRPSQG	1	2	1	P02858		0.0000	0	0.001727	47	0.009999094	3	2458.02976	2.42	13.95
**High**	WQEQQDEDEDE	1	2	1	P02858		0.0000	0	0.0003587	44	0.002887334	2	1450.52458	0.30	16.65
**High**	DEQIPSHPPR	1	2	1	P02858		0.0000	0	0.006591	37	0.572071635	2	1175.58232	1.63	15.86
**High**	DEDEDDEDEQIPSHPPRRPSHG	1	2	1	P02858		0.0000	0	1.509E-08	36	0.169319411	4	2557.08559	1.28	16.00
**High**	EQDEDEDEDEDKPRPSRPS	1	2	1	P02858		0.0000	0	0.0002238	33	0.296062992	3	2272.94785	1.80	14.01
**High**	QGQHQQEEEEEGGSV	1	2	1	P02858		0.0000	0	0.002631	27	1.37252143	2	1670.69341	2.68	14.27
**High**	KEQQQEQQQEEQPLE	1	1	1	P0DO15		0.0000	0	0.000009733	74	9.53075E-05	2	1898.87700	2.26	15.63
**High**	IGINAENNQRN	1	1	1	P0DO15		0.0000	0	0.01234	67	0.000469711	2	1242.61943	0.68	15.67
**High**	LFSREEGQQQGEQ	1	1	1	P0DO15		0.0000	0	0.01571	61	0.001976832	2	1535.71123	1.75	15.96
**High**	SREEGQQQGEQRLQE	2	1	1	P0DO15		0.0000	0	7.355E-08	59	0.003467165	3	1801.84696	2.52	14.14
**High**	FSREEGQQQGEQ	2	1	1	P0DO15		0.0000	0	0.002445	59	0.002160545	2	1422.62663	1.52	13.31
**High**	KEQQQEQQQEEQPL	1	1	1	P0DO15		0.0000	0	0.01296	58	0.004813334	2	1769.83599	3.32	15.84
**High**	EQQQEQQQEEQPLE	1	1	1	P0DO15		0.0000	0	0.0003122	54	0.005880889	2	1770.78081	1.73	16.99
**High**	SLVNNDDRDS	3	1	1	P0DO15		0.0000	0	0.05486	48	0.026660789	2	1134.50237	0.13	16.34
**High**	SREEGQQQGEQRLQES	1	1	1	P0DO15		0.0000	0	0.00000703	46	0.063416736	3	1888.87552	0.57	14.20
**High**	VGLKEQQQEQ	1	1	1	P0DO15		0.0000	0	0.0264	44	0.091111039	2	1186.60820	1.61	14.25
**High**	LKEQQQEQQQEEQPLE	2	1	1	P0DO15		0.0000	0	0.00008758	39	0.42622471	2	2011.96257	2.88	16.58
**High**	VGLKEQQQEQQ	1	1	1	P0DO15		0.0000	0	0.02885	37	0.542579939	2	1314.66655	1.28	14.36
**High**	QREEQEWPRKEEK	1	1	1	P0DO15		0.0000	0	0.00003053	25	10.75979545	4	1771.87368	0.79	13.47

**High**	PQHPEREPQQPGEKEEDE	1	1	1	P0DO15		0.0000	0	0.001634	24	6.904048974	3	2158.96762	1.83	13.66
**High**	FGREEGQQQGEE	1	1	1	P11827		0.0000	0	0.002668	60	0.001233267	2	1393.59844	0.36	14.21
**High**	GREEGQQQGEERLQE	2	1	1	P11827		0.0000	0	3.254E-08	59	0.002900604	3	1772.81980	2.21	14.58
**High**	FVDAQPQQKEEGN	3	1	1	P11827		0.0000	0	0.008778	59	0.00288809	2	1489.69463	1.88	15.57
**High**	FVDAQPQ	1	3	2	P11827;F7J077		0.0000	0	0.004484	51	0.023412219	2	804.38951	1.03	16.56
**High**	GREEGQQQGEERLQES	1	1	1	P11827		0.0000	0	1.368E-07	50	0.020554019	3	1859.85184	2.11	14.59
**High**	FVDAQPQQKEEG	1	3	2	P11827;F7J077		0.0000	0	0.001301	48	0.039871276	2	1375.65141	1.83	15.56
**High**	TLVNNDDRDS	2	3	2	P11827;F7J077		0.0000	0	0.003046	45	0.057454064	2	1148.51921	1.17	14.72
**High**	DEEQDKESQESEGSESQREPR	2	1	1	P11827		0.0000	0	0.000002046	39	0.074475433	4	2479.04726	0.81	13.72
**High**	EREHPRPHQPHQKEEEKHE	2	1	1	P11827		0.0000	0	0.00008161	34	1.664037557	4	2457.18374	2.85	8.26
**High**	ERQQHGEKEEDEGEQPRP	1	1	1	P11827		0.0000	0	3.237E-07	31	1.395967223	4	2177.98086	0.06	12.24
**High**	EEEDQDEDEEQDKESQESEGSESQREPR	1	1	1	P11827		0.0000	0	0.000007023	27	0.226686836	4	3353.33632	2.44	14.76
**High**	REHPRPHQPHQKEEEKHE	1	1	1	P11827		0.0000	0	8.73251E-09	26	10.51995116	5	2328.14069	2.82	8.23
**High**	EQDKESQESEGSESQREPR	1	1	1	P11827		0.0000	0	0.04485	23	6.231339126	3	2234.97910	1.52	12.34
**High**	DEREHPRPHQPHQKEEEKHE	1	1	1	P11827		0.0000	0	0.0001336	16	82.67284865	4	2572.21938	6.10	8.38
**High**	QPHQEEEHEQKEEHEWHRKEE	1	1	1	P11827		0.0000	0	0.02159	16	34.39622416	5	2808.23835	0.92	13.10
**High**	HGGKGSEEEQDEREHPRPHQPHQKEEEKHE	2	1	1	P11827		0.0000	0	0.002019	14	92.62774546	7	3610.64238	0.55	8.53
**High**	HEQKEEHEWHRKEE	1	1	1	P11827		0.0000	0	0.05024	12	167.7327612	4	1930.88540	3.24	8.47
**High**	VVAEQGGEQGLE	2	1	1	A0A0R0GMV1		0.0000	0	5.523E-07	82	1.49779E-05	2	1215.58672	1.21	17.41
**High**	NNQLDQNPR	3	1	1	A0A0R0GMV1		0.0000	0	0.02175	59	0.002769075	2	1098.52983	1.03	13.71
**High**	VAEQGGEQGLE	1	1	1	A0A0R0GMV1		0.0000	0	0.003627	57	0.00395427	2	1116.51775	0.83	16.44
**High**	NALEPDHRVESEGG	1	3	2	P02858; A0A0R0GMV1		0.0000	0	0.005443	49	0.031992697	2	1509.69512	1.47	15.69
**High**	AGNPDIEHPET	1	1	1	A0A0R0GMV1		0.0000	0	0.03474	45	0.053652047	2	1179.52861	0.76	16.24
**High**	FVDAQPQQKEEGSKG	1	2	1	F7J077		0.0000	0	1.466E-08	67	0.000658928	2	1647.80388	3.95	14.47
**High**	FVDAQPQQKEEGS	1	2	1	F7J077		0.0000	0	0.01407	53	0.011572444	2	1462.68462	2.52	15.54
**High**	FVDAQPQ	1	3	2	P11827;F7J077		0.0000	0	0.004484	51	0.023412219	2	804.38951	1.03	16.56
**High**	FVDAQPQQKEEG	1	3	2	P11827;F7J077		0.0000	0	0.001301	48	0.039871276	2	1375.65141	1.83	15.56

**High**	TLVNNDDRDS	2	3	2	P11827;F7J077		0.0000	0	0.003046	45	0.057454064	2	1148.51921	1.17	14.72
**High**	QRIPAGTT	1	2	1	F7J077		0.0000	0	0.01148	31	1.315264888	2	843.47093	3.09	14.46
**High**	LKVREDENNP	1	2	1	F7J077		0.0000	0	0.05486	30	2.954393028	2	1213.61785	0.53	14.19
**High**	IMSDESTESETEQ	1	2	1	O64458		0.0000	0	0.000002172	88	6.47571E-07	2	1485.59160	1.22	16.37
**High**	IMSDESTESETEQA	1	2	1	O64458		0.0000	0	0.00009654	63	0.000252387	2	1556.62859	1.08	17.15
**High**	YQEPQESQQRG	1	1	1	P04405		0.0000	0	0.0009055	55	0.006528817	2	1349.61199	2.90	14.13
**High**	YLAGNQEQE	17	2	2	P04776;P04405		0.0000	0	0.0109	54	0.007138189	2	1051.46929	0.17	22.14
**High**	RNLQGENEEEDSGA	1	1	1	P04405		0.0000	0	0.0000809	52	0.005817199	2	1547.66057	2.36	14.54
**High**	MRKPQQEEDDDDE	1	1	1	P04405		0.0000	0	0.02409	41	0.03625453	2	1634.66533	3.28	12.65
**High**	RQNIGQNSSPD	2	1	1	P04405		0.0000	0	0.02188	41	0.180812407	2	1215.57109	-0.17	13.18
**High**	FNENESGDQV	1	1	1	P05046		0.0000	0	0.006334	61	0.000571086	2	1138.46489	0.11	17.17
**High**	NKVDENGTPKPS	4	1	1	P05046		0.0000	0	0.002978	57	0.005876098	2	1285.63957	0.97	12.00
**High**	GGTGGTDYGTTGGTG	1	1	1	K7LEQ5		0.0000	0	0.0057	78	1.279E-05	2	1258.52141	2.49	15.19
**High**	YGNVEKQTDE	1	1	1	K7LEQ5		0.0000	0	0.0003858	47	0.030024099	2	1182.52995	2.19	14.13
**High**	YGTNTADTGTGPR	1	1	1	K7LEQ5		0.0000	0	0.001024	41	0.187711656	2	1310.60063	2.63	14.68
**High**	NLQGENEGEDKGA	1	1	1	P04776		0.0000	0	0.007443	54	0.005853614	2	1360.59941	1.33	13.99
**High**	YLAGNQEQE	17	2	2	P04776;P04405		0.0000	0	0.0109	54	0.007138189	2	1051.46929	0.17	22.14
**High**	IKPPTDEQQQRPQE	2	1	1	P04776		0.0000	0	0.0218	41	0.273058321	2	1693.85539	2.91	15.43
**High**	VIKPPTDEQQQRPQE	1	1	1	P04776		0.0000	0	0.00006892	39	0.449881374	3	1792.91965	0.43	15.98
**High**	IKPPTDEQQQRPQEE	1	1	1	P04776		0.0000	0	0.0004184	32	2.554844876	3	1822.89640	1.83	15.51
**High**	VIKPPTDEQQQRPQEE	1	1	1	P04776		0.0000	0	0.000217	28	5.829024329	3	1921.96305	0.81	16.17
**High**	IKPPTDEQQQRPQEEEE	1	1	1	P04776		0.0000	0	0.001827	25	9.864223021	3	2080.98337	2.46	15.83
**High**	GKHQQEEENEGGSI	1	1	1	P04776		0.0000	0	0.04737	23	8.540962679	2	1541.68645	2.42	13.54
**High**	IKPPTDEQQQRPQEEE	1	1	1	P04776		0.0000	0	0.03119	20	34.28549271	3	1951.93778	1.08	15.76
**High**	QAALQTPK	1	3	1	I1L860		0.0000	0	0.04134	42	0.075457449	2	856.48949	0.88	15.05
**High**	QAALQTPKG	1	3	1	I1L860		0.0000	0	0.02992	31	1.144109738	2	913.51079	0.64	15.38

Table S4. Residues involved in the binding of Keap1 with the analyzed peptides. The residues involved in the binding between Keap1 and Nrf2 are highlighted in red

Keap 1	N-14-G	K-14-L	H-14-E	M-13-E	G-14-I	D-10-R	S-10-S	F-12-G	V-11-R	I-11-N	F-12-E	Y-9-E
**Y 334**	H7	E2					N5	P6	R11			
**R 336**			E5								K9	
**S 363**			R11		G12					Q9	E10	
**R 380**	E10, E12	L14	K4	E8	E10	S6	D9	D3, Q5, Q8			E10	Q7, E9
**N 382**			E5			H7		Q5		R10	Q8	
**D 385**			Q3							R10		
**N 387**	E12		H1, E5					D3				
**D 389**			Q3	Q6	K2							
**N 414**	R8	E6	E14		E10		R8					
**R 415**	D6	E6	E8, E14	D9, D11		E2	N5, D7	D3, G12	Q7	E6, N11	F1, Q5, E12	E9
**S 431**		E6		E7								
**H 432**	G 14											
**H 436**				E7								
**R 483**		E10; E11	E8	D9, D10				E11	E4	E6	F2	
**S 508**										E6		
**Y 525**							V3				D3	
**Q 530**								E10		E6		
**Y 572**					E6				R11		K9	
**S 602**	D6	Q12	R11	D12	S13					N7	G10	

Table S5. ABTS assay with identified soy peptides (0.05 mg/mL). Values were indicated as TEAC (µM Trolox)

Peptide	TEAC (µM Trolox)
**N-14-G**	0.12 ± 0.22
**K-14-L**	0.34 ± 0.20
**H-14-E**	0.63 ± 0.27
**M-13-E**	0.25 ± 0.13 (***)
**G-14-I**	0.14 ± 0.43
**D-10-R**	n.d.
**S-10-S**	n.d.
**F-12-G**	n.d.
**V-11-R**	n.d.
**I-11-N**	n.d.
**F-12-E**	n.d.
**Y-9-E**	n.d.
**F-12-Q**	n.d.

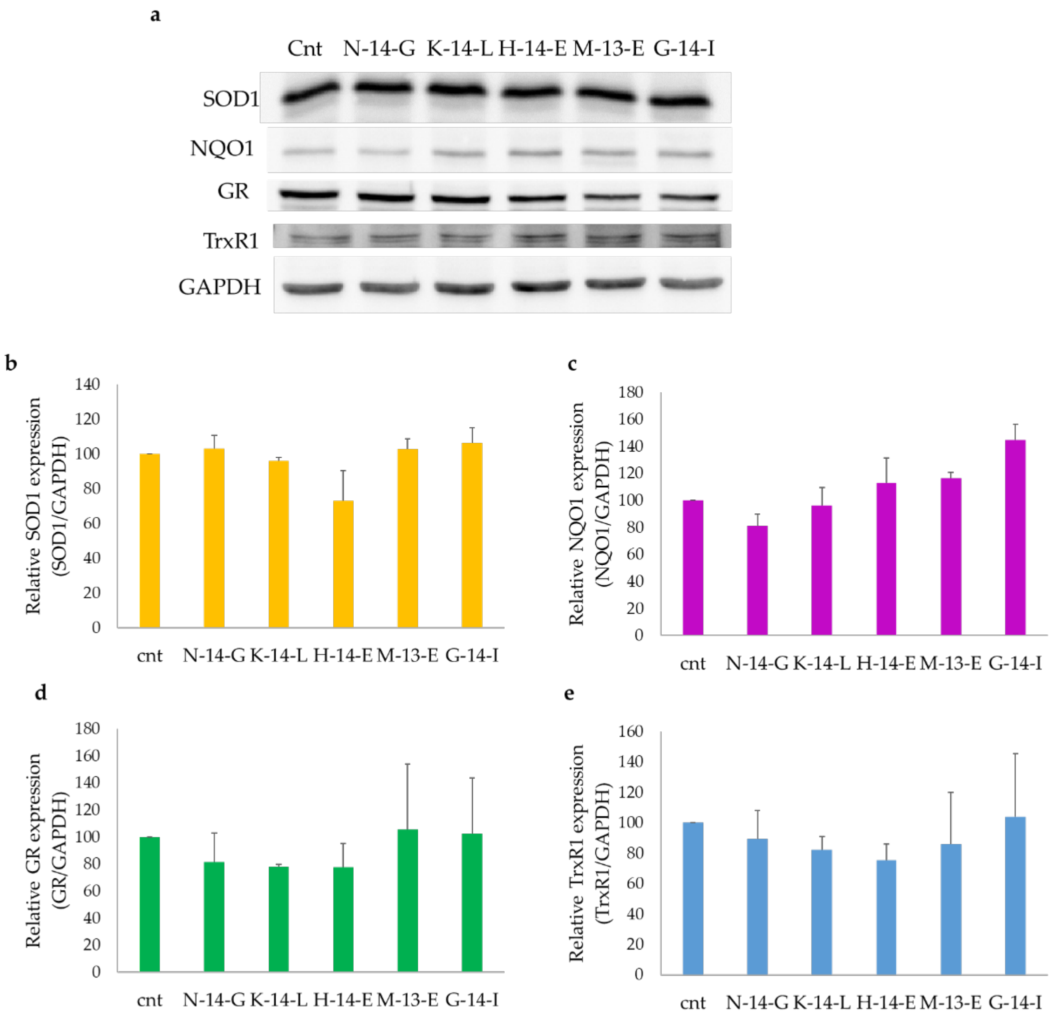

Figure S3. Western Blot analysis of antioxidant and phase II enzymes in Caco-2 cell lysates treated with **N-14-G**, **K-14-L**, **H-14-E**, **M-13-E** and **G-14-I** (0.05 mg/mL) for 24 h (**a**). **b-e)** Quantification of the above reported Western Blot.

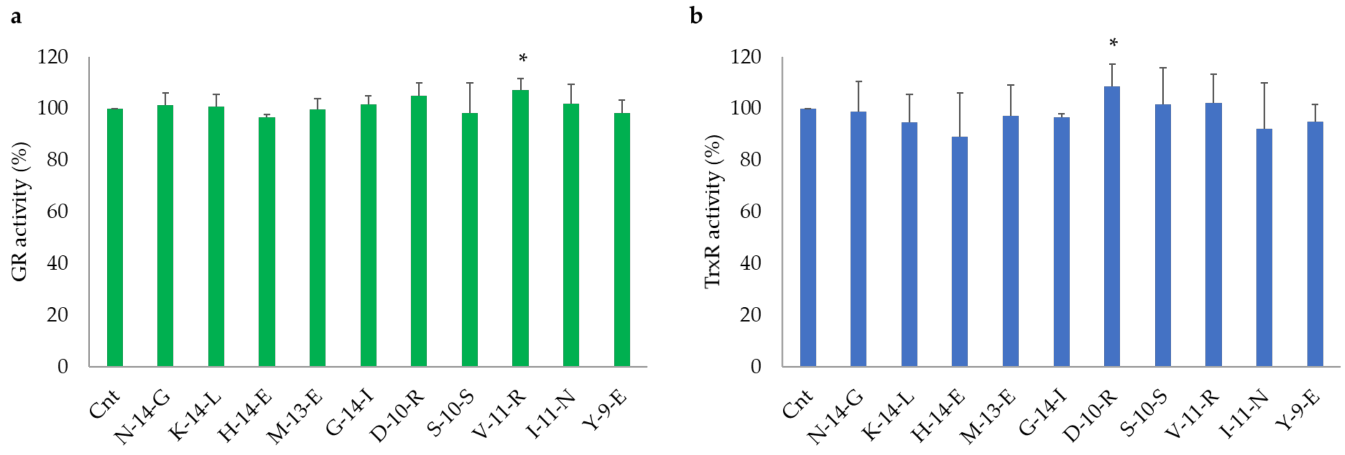

Figure S4. GR (**a**) and TrxR activity (**b**) in Caco-2 cell lysates treated with soy peptides (0.05 mg/mL) for 24 h.

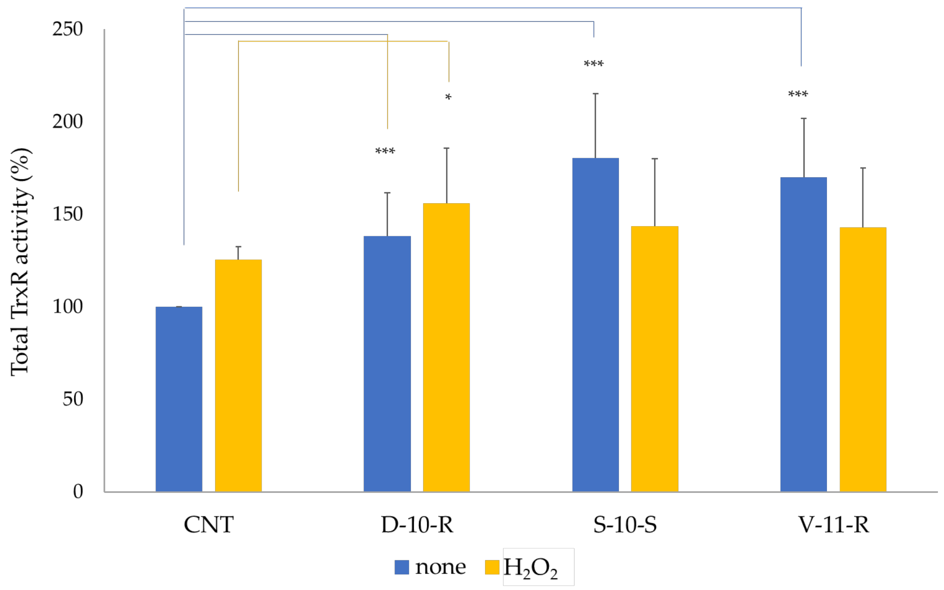

Figure S5. Total TrxR activity (%) in Caco-2 cells treated with **D-10-R**, **S-10-S** and **V-11-R** (0.05 mg/mL) in the presence or absence of 2.5 mM H_2O_2 for 48 h.
